# Andrias Davidianus Peptide Hydrogel Enables Sustained SR9011 Release to Promote Efferocytosis and Alleviate Colitis

**DOI:** 10.1002/smll.202509049

**Published:** 2025-10-30

**Authors:** Yidong Chen, Jiamin Li, Junrong Li, Xiaopeng Zhang, Fang Liu, Qi Yu, Rong Lin, Liangru Zhu

**Affiliations:** ^1^ Division of Gastroenterology Union Hospital Tongji Medical College Huazhong University of Science and Technology Wuhan 430022 China; ^2^ Department of Endoscopy and Digestive System Guizhou Provincial People's Hospital Guiyang 550000 China

**Keywords:** CD47, efferocytosis, hydrogel drug delivery, NR1D1, SR9011, ulcerative colitis

## Abstract

Ulcerative colitis (UC) is a chronic inflammatory disorder marked by epithelial barrier disruption and defective resolution of inflammation. Although epithelial apoptosis and impaired efferocytosis are recognized contributors to disease progression, their molecular regulation remains insufficiently defined. NR1D1, a circadian transcriptional repressor implicated in immune control, has not been fully explored in UC. Here, this study shows that NR1D1 expression is markedly reduced in human UC biopsies and murine colitis. CUT&Tag profiling revealed direct NR1D1 chromatin occupancy, while ChIP–qPCR and luciferase assays confirmed that NR1D1 represses CD47, a key anti‐efferocytosis molecule. Functional experiments demonstrate that NR1D1 deficiency enhances epithelial apoptosis, upregulates CD47, impairs macrophage efferocytosis, and exacerbates colitis in IEC‐specific *Nr1d1^−/−^* mice. To restore NR1D1 activity, a hydrogel (HA@Alg‐ADAP‐SR9011) combining Andrias davidianus peptides is engineered with the NR1D1 agonist SR9011 for targeted, sustained intestinal delivery. In DSS‐induced colitis, hydrogel‐mediated SR9011 suppresses inflammation, reduces CD47, preserves epithelial integrity, and improves histological outcomes. Single‐cell RNA sequencing further reveals reduced CD47 and enhances efferocytic signatures in SR9011‐treated tissues. While pharmacokinetic analyses are limited to colonic tissue, these findings establish NR1D1 as a central regulator of epithelial turnover and efferocytosis in UC and support hydrogel‐based NR1D1 activation as a therapeutic strategy.

## Graphical abstract

NR1D1 suppresses epithelial CD47 transcription to promote efferocytosis by macrophages. In UC, NR1D1 expression is diminished, leading to CD47 upregulation, inhibition of apoptotic cell clearance, and exacerbation of intestinal inflammation. Restoring rhythmic NR1D1 activation using SR9011‐loaded bioadhesive hydrogel enhances efferocytosis, improves epithelial homeostasis, and ameliorates colitis severity.

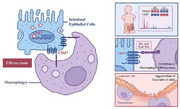

## 1 Introduction

Ulcerative colitis (UC) is a chronic, relapsing inflammatory disease of the colonic mucosa characterized by alternating periods of remission and flare,^[^ ^1^, ^2^ ^]^ with clinical manifestations ranging from mild discomfort to life‐threatening complications such as toxic megacolon and colorectal cancer.^[^ ^3^ ^]^ Persistent mucosal inflammation disrupts the balance between immune tolerance and activation, driving epithelial injury, ulceration, and barrier dysfunction,^[^ ^4^ ^]^ and despite therapeutic advances including biologics and immunomodulators, the molecular mechanisms underlying UC pathogenesis remain incompletely understood.^[^ ^5^ ^]^ Central to disease progression is a dysregulated immune response, fuelled by aberrant interactions between the host immune system and the intestinal microenvironment.^[^ ^6^ ^]^ The intestinal epithelium is pivotal to gut homeostasis, acting both as a physical barrier against luminal microbes and as an active modulator of mucosal immunity.^[^ ^7^, ^8^ ^]^ In UC, epithelial injury increases intestinal permeability, enabling microbial antigens to penetrate the lamina propria and trigger exaggerated immune responses^[^ ^9^ ^]^ which amplify the release of inflammatory mediators, further damage the epithelium, and sustain mucosal inflammation.^[^ ^10^ ^]^ Beyond barrier protection, epithelial cells also secrete cytokines and chemokines that shape immune cell behavior and maintain tolerance to commensal microbes^[^ ^11^ ^]^; loss of this regulatory function contributes to chronic inflammation and impairs the restoration of mucosal integrity.^[^ ^12^, ^13^ ^]^

In UC, efferocytosis is impaired, leading to defective clearance of apoptotic epithelial cells and their pathological accumulation.^[^ ^14^, ^15^, ^16^ ^]^ These uncleared cells undergo secondary necrosis, releasing intracellular contents that amplify mucosal inflammation and impede tissue repair. Efferocytosis—the process by which macrophages engulf apoptotic cells—is normally integral to the resolution of inflammation and the restoration of tissue homeostasis.^[^ ^17^, ^18^ ^]^ This clearance depends on a finely tuned balance between pro‐engulfment “eat me” signals (e.g., phosphatidylserine) and inhibitory “don't eat me” signals (e.g., CD47 engaging macrophage SIRPα) that restrain phagocytosis.^[^ ^19^, ^20^ ^]^ When macrophage efferocytic function is impaired in UC, unresolved inflammation exacerbates epithelial injury and disrupts the regenerative processes required to restore gut integrity.^[^ ^21^ ^]^

The circadian clock is an intrinsic timekeeping system that regulates metabolism, immune responses, and cellular repair through transcriptional–translational feedback loops, in which CLOCK–BMAL1 activates PER and CRY, whose accumulation represses CLOCK–BMAL1 to generate ≈24 h rhythmic oscillations.^[^ ^22^, ^23^ ^]^ Nuclear receptors such as NR1D1 (REV‐ERBα) and ROR fine‐tune this loop by controlling BMAL1 transcription, thereby ensuring robust circadian precision.^[^ ^24^ ^]^ Beyond temporal regulation, the circadian clock profoundly influences mucosal immunity and intestinal homeostasis.^[^ ^25^ ^]^ Disruption of circadian rhythms, common in modern lifestyles, has been implicated in chronic inflammatory diseases, including UC.^[^ ^26^ ^]^ Among core regulators, NR1D1 has emerged as a critical link between circadian and immune control.^[^ ^27^ ^]^ Acting as a transcriptional repressor, NR1D1 suppresses pro‐inflammatory gene expression and modulates metabolic and immune pathways, particularly in macrophages; its downregulation during intestinal inflammation suggests a pathogenic role in UC.^[^ ^28^, ^29^ ^]^ Because NR1D1 oscillates diurnally—peaking by day and declining at night—we sought to avoid bolus administration of NR1D1 agonists that could disrupt its rhythm. Instead, we engineered a bioadhesive hydrogel composed of Andrias davidianus active peptides (ADAP) and alginate, designed to encapsulate and sustain release of SR9011, a synthetic NR1D1 agonist. ADAP‐based hydrogels offer biocompatibility, intrinsic bioactivity, and strong mucosal adhesion,^[^ ^30^, ^31^ ^]^ enabling rhythmic NR1D1 activation with prolonged colonic retention. Targeted drug delivery, already proven effective across multiple therapies, offers a promising means to restore immune and epithelial homeostasis in UC.^[^ ^32^, ^33^, ^34^ ^]^

In this study, we explored the therapeutic potential of NR1D1 activation in UC, hypothesizing that sustained stimulation could mitigate mucosal injury and promote repair. We developed an ADAP‐based hydrogel for controlled SR9011 delivery, aligning with circadian rhythms, to restore intestinal homeostasis through combined regulation of inflammation, efferocytosis, and epithelial integrity.

## 2 Results

### 2.1 NR1D1 Coordinates Epithelial Apoptosis and Efferocytosis via CD47 Repression in UC

**Figure 1:**
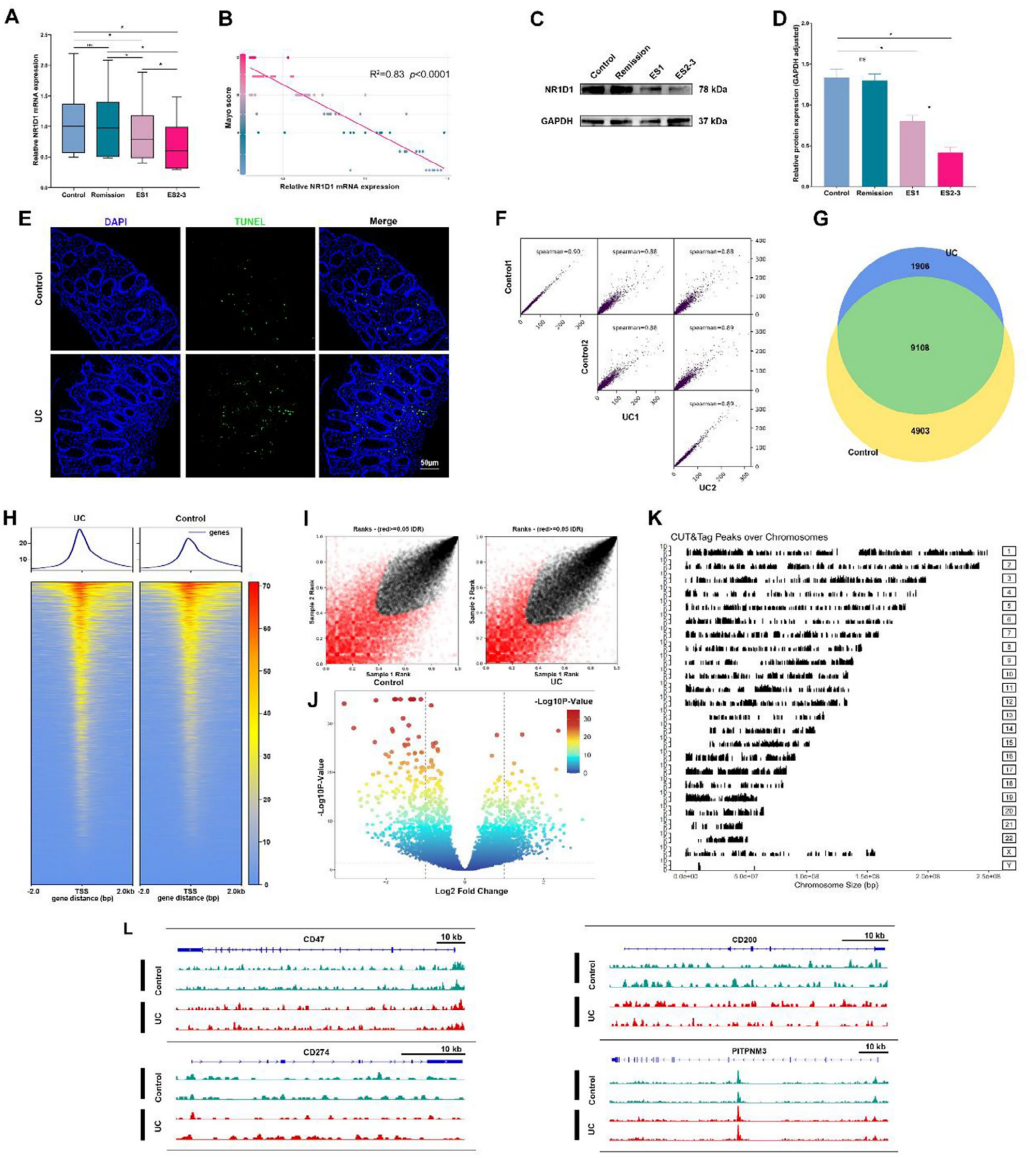
NR1D1 expression is downregulated in UC and displays altered chromatin occupancy. A) Relative *NR1D1* mRNA levels in colonic mucosa from healthy controls, UC patients in remission, and patients with mild (ES1) or moderate–severe (ES ≥2–3) endoscopic activity (one‐way ANOVA). B) Inverse correlation between NR1D1 mRNA expression and Mayo endoscopic subscores (R^2^ = 0.83, *P* < 0.0001). C) Representative immunoblot of NR1D1 protein across UC stages, with GAPDH as loading control. D) Quantification of immunoblot data normalized to GAPDH (*n* = 12; one‐way ANOVA). E) TUNEL staining showing increased epithelial apoptosis in UC compared with controls (*n* = 6). F) Pairwise Spearman correlation of CUT&Tag replicates (Control1/2, UC1/2) demonstrating high reproducibility. G) Venn diagram of NR1D1 binding peaks showing shared and condition‐specific sites. H) Heatmaps and peak distribution plots depicting NR1D1 binding around transcription start sites (TSS ±2 kb). I) Irreproducible discovery rate (IDR) analysis confirming reproducibility across replicates. J) Volcano plot of differential NR1D1 binding between UC and controls, highlighting significant gains and losses. K) Genome‐wide distribution of NR1D1 CUT&Tag peaks. L) Representative IGV tracks showing NR1D1 occupancy at the CD47, CD74, CD200, and PTPRM loci. Data are presented as mean ± S.D.; ns, not significant; ^*^ *p* <0.05.

To determine the clinical relevance of NR1D1 in UC, we analyzed colonic mucosal biopsies from patients and controls, collected between 9:00 and 11:00 AM to minimize diurnal variation. *NR1D1* mRNA was markedly reduced in active UC, particularly in patients with higher Mayo endoscopic scores, but remained comparable to controls in remission (**Figure** **1**A,B). Protein analyses confirmed diminished NR1D1 levels in active UC, with the greatest reductions in severe cases (Figure 1C,D). TUNEL staining further revealed increased epithelial apoptosis in UC tissues, suggesting a link between NR1D1 loss and impaired epithelial homeostasis (Figure 1E).

To determine whether NR1D1 deficiency promotes epithelial death through heightened apoptosis, defective clearance, or both, we profiled NR1D1 chromatin occupancy using CUT&Tag (Tn5‐mediated) in colonic biopsies from UC patients and controls. Replicates were highly consistent (Figure 1F). Comparative peak analysis revealed extensive overlap between groups alongside condition‐specific sites, indicating remodeling of NR1D1 binding in UC (Figure 1G). Binding was enriched near transcription start sites in both groups (Figure 1H), and reproducibility was confirmed by irreproducible discovery rate analysis (Figure 1I). Differential peak analysis showed marked changes in NR1D1 occupancy (Figure 1J), with enrichment of pathways related to inflammation and apoptosis (Figure S1A–D, Supporting Information). Genome‐wide mapping demonstrated broad chromosomal distribution, with clear occupancy near the CD47 locus (Figure 1K,L).

ChIP–qPCR in human colonic organoids and isolated mouse intestinal epithelial cells (IECs) confirmed NR1D1 enrichment at the CD47 promoter (Figure S2A–C, Supporting Information). NR1D1 knockdown upregulated CD47 expression, whereas CD47 knockdown had no reciprocal effect, establishing a unidirectional regulatory link (Figure S2D–I, Supporting Information). Promoter–reporter assays further showed that NR1D1 directly represses CD47 transcription, an effect abolished by mutation of NR1D1‐binding motifs (Figure S2J,K, Supporting Information).

**Figure 2:**
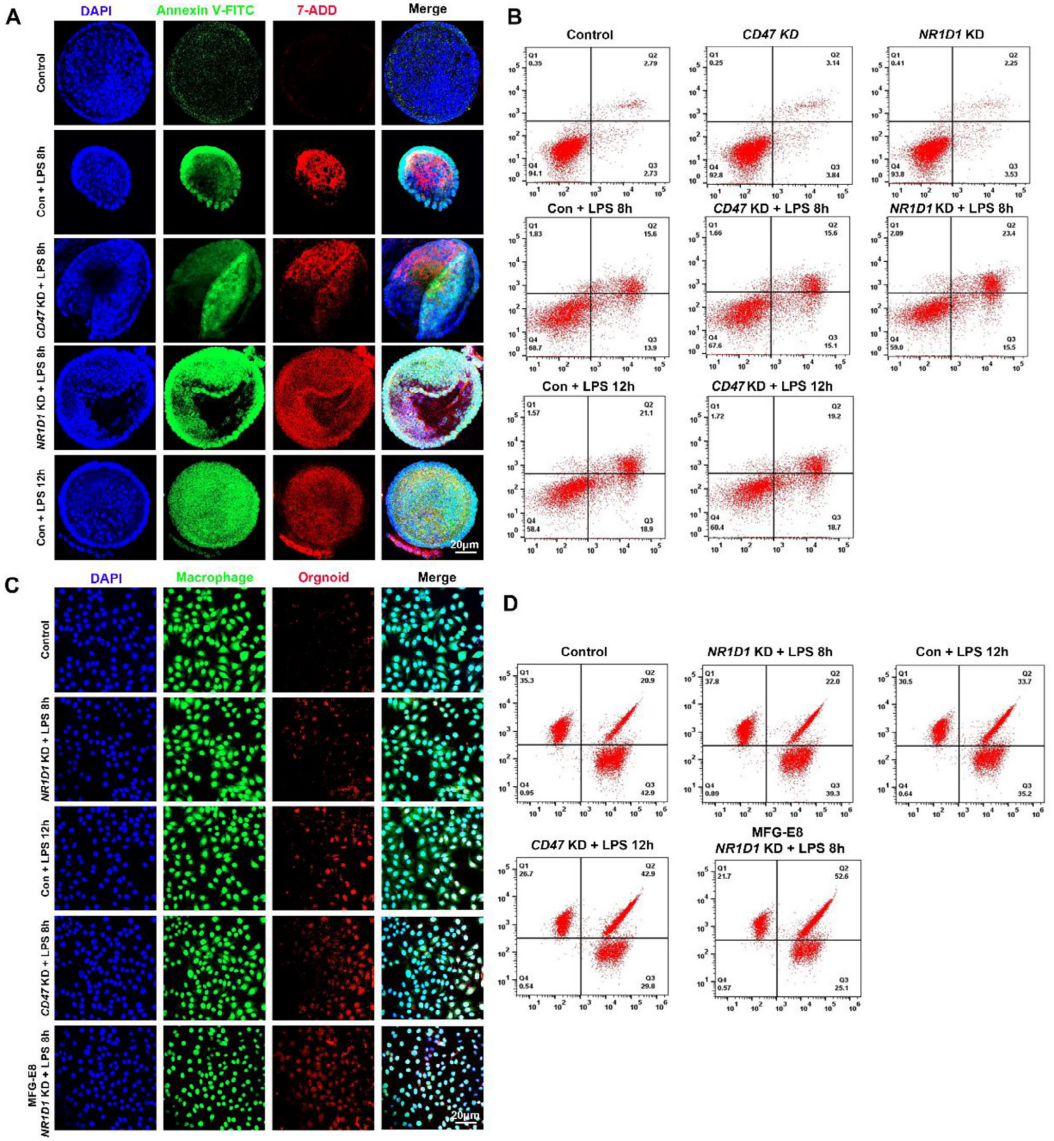
NR1D1 deficiency promotes epithelial apoptosis and impairs macrophage efferocytosis via CD47 regulation. A) Confocal images of colonic organoids stained with Annexin V–FITC (green) and 7‐AAD (red) under the indicated conditions. NR1D1 knockdown (KD) increased apoptosis after LPS stimulation compared with control or CD47 KD. B) Flow cytometry of Annexin V/7‐AAD staining confirmed elevated apoptosis in NR1D1 KD organoids after 8 h of LPS, comparable to 12 h controls; CD47 KD showed no effect. C) Confocal images of macrophage–organoid co‐cultures. Organoids were labeled with CMTPX (red), macrophages with CMFDA (green), and nuclei with DAPI (blue). NR1D1 KD impaired efferocytosis, whereas CD47 KD and MFG‐E8 treatment enhanced uptake. D) Flow cytometry plots of macrophage uptake of apoptotic organoids under the indicated conditions, showing reduced efferocytosis with NR1D1 KD and enhanced clearance with CD47 KD or MFG‐E8. Representative of six independent experiments (*n* = 6).

To examine the NR1D1–CD47 axis, we co‐cultured macrophages with LPS‐treated colonic organoids under control, CD47 knockdown, and NR1D1 knockdown conditions. NR1D1 silencing accelerated epithelial apoptosis, with cells reaching late‐stage death several hours earlier than controls, whereas CD47 knockdown had no effect (**Figure** **2**A,B). Efferocytosis assays with fluorescently labeled organoids and macrophages showed that NR1D1 deficiency impaired clearance, while CD47 knockdown enhanced uptake; recombinant MFG‐E8 restored efferocytosis in NR1D1‐deficient settings (Figure 2C,D). Comparable results were obtained when apoptosis was induced with staurosporine in the absence of inflammation (Figure S3A, Supporting Information). Notably, dual knockdown of NR1D1 and CD47 further increased efferocytosis compared with CD47 knockdown alone, underscoring the functional interplay between these regulators (Figure S3B,C, Supporting Information). Together, these findings demonstrate that NR1D1 loss promotes UC pathology by accelerating epithelial apoptosis and suppressing efferocytosis through derepression of CD47.

### 2.2 Epithelial NR1D1 Deficiency Exacerbates Colitis by Enhancing Apoptosis and Impairing Efferocytosis

To assess the role of NR1D1 in intestinal inflammation, we generated IEC‐specific *Nr1d1* knockout mice (*Nr1d1* ^−/−^) and induced colitis with dextran sulfate sodium (DSS). Under steady‐state conditions, NR1D1 loss caused no overt mucosal abnormalities, but following DSS challenge, knockout mice showed markedly exacerbated colonic injury compared with wild‐type (WT) controls (Figure S4A, Supporting Information). Immunofluorescence confirmed loss of NR1D1 in colonic epithelium (Figure S4B, Supporting Information). DSS‐treated *Nr1d1* ^−/−^ mice exhibited shorter colons, higher inflammation scores, increased disease activity index, and greater weight loss (Figure S4C–F, Supporting Information).

Cytokine profiling revealed elevated TNF‐α, IL‐1β, and IL‐17, together with reduced IL‐10, indicating a shift toward a pro‐inflammatory epithelial milieu (Figure S5A–D, Supporting Information). Immunofluorescence of tight‐junction proteins ZO‐1 and occludin showed reduced expression in *Nr1d1* ^−/−^ colons during colitis, consistent with impaired barrier integrity (Figure S5E,F, Supporting Information).

**Figure 3:**
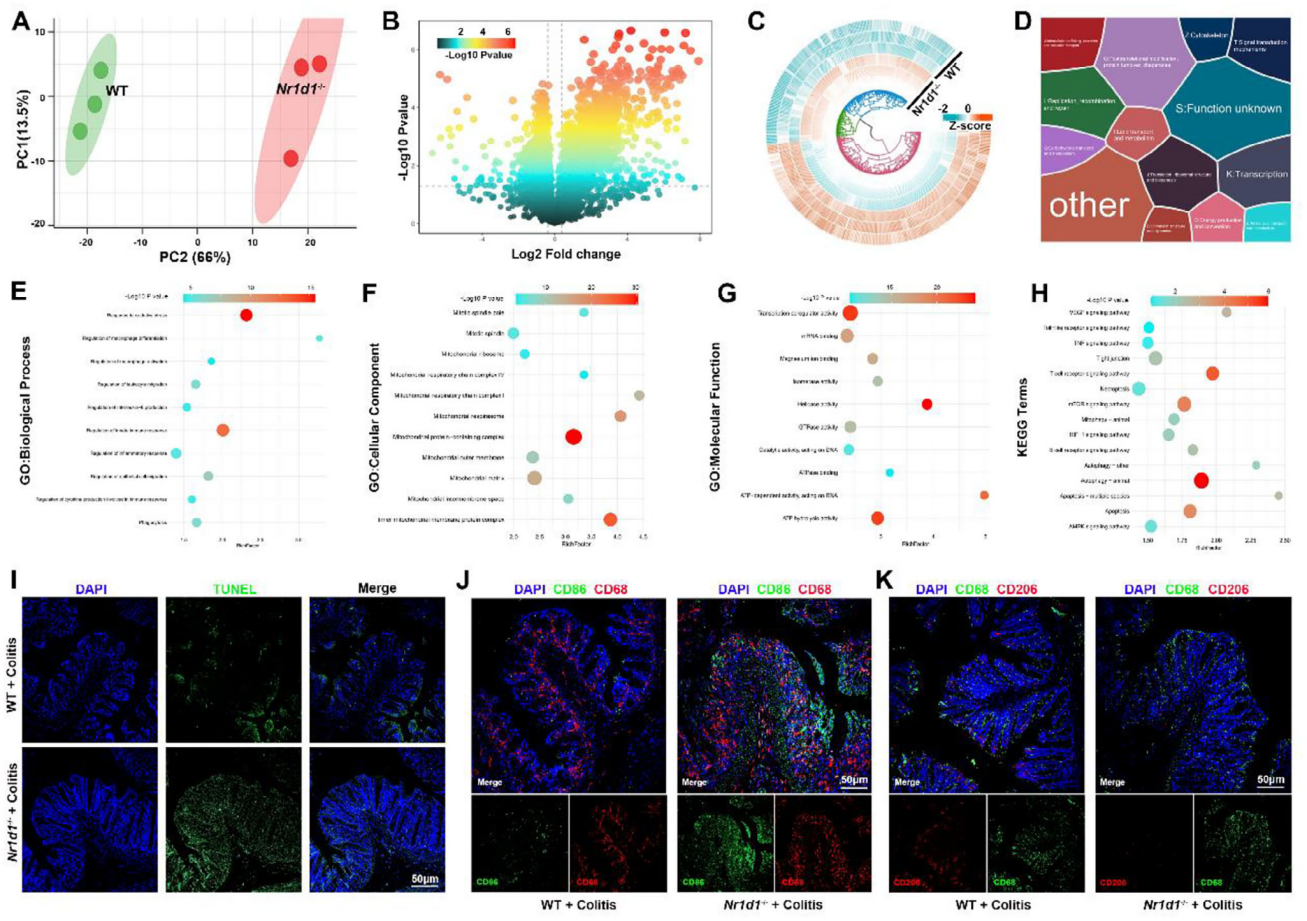
NR1D1 deficiency exacerbates epithelial apoptosis, skews macrophage polarization, and alters inflammation‐associated transcriptional programs in colitis. A) Principal component analysis (PCA) of proteomic profiles from IECs of WT and *Nr1d1^−/−^* mice with DSS‐induced colitis, showing distinct group separation (*n* = 3). B) Volcano plot of differentially expressed proteins between WT and *Nr1d1^−/−^* colitis mice (*n* = 3). C) Hierarchical clustering heat map of protein Z‐scores (*n* = 3). D) Functional annotation of protein clusters based on Clusters of Orthologous Groups (COG). (E–G) Gene Ontology (GO) enrichment analyses of biological processes (E), cellular components (F), and molecular functions (G) altered in Nr1d1‐/‐ mice (*n* = 3). H) KEGG pathway analysis highlighting enrichment of apoptosis, TNF, NF‐κB, and tight junction signaling, consistent with enhanced inflammation and epithelial dysfunction in the *Nr1d1^−/−^* group (*n* = 3). I) TUNEL staining of colon sections showing increased epithelial apoptosis in *Nr1d1^−/−^* versus WT controls (*n* = 6). J) Immunofluorescence staining of CD86 (green) and CD68 (red) showing increased pro‐inflammatory M1 macrophages in *Nr1d1^−/−^* colitis mice (*n* = 6). K) Immunofluorescence staining of CD206 (red) and CD68 (green) showing reduced anti‐inflammatory M2 macrophages in *Nr1d1^−/−^* colitis mice (*n* = 6).

Proteomic profiling of IECs from *Nr1d1* ^−/−^ and WT mice with colitis revealed substantial differences in protein expression. Principal component analysis (PCA) showed clear separation between genotypes (**Figure** **3**A), supported by a differential expression volcano plot (Figure 3B) and circular heatmap (Figure 3C). Voronoi mapping of identified proteins (Figure 3D), along with GO and KEGG enrichment analyses, highlighted pathways involved in apoptosis, inflammation, and phagocytosis (Figure 3E–H), suggesting that NR1D1 deficiency alters key regulatory networks associated with epithelial integrity and immune resolution.

To assess epithelial apoptosis, we performed TUNEL staining on colonic tissues, which revealed significantly increased apoptotic cell numbers in *Nr1d1* ^−/‐^ mice during colitis (Figure 3I). Furthermore, immunofluorescence analysis of macrophage subtypes indicated a skewed polarization in *Nr1d1* ^−/−^ mice, with increased infiltration of pro‐inflammatory M1 macrophages (CD68⁺CD86⁺) and a concomitant reduction in anti‐inflammatory, pro‐efferocytic M2 macrophages (CD68⁺CD206⁺) (Figure 3J,K). Quantification confirmed a significantly reduced M2/M1 ratio in *Nr1d1* ^−/−^ colitic mice (Figure S6A–D, Supporting Information). Given that M2 macrophages possess greater efferocytic capacity, whereas M1 macrophages are inefficient in clearing apoptotic cells and can perpetuate inflammation, these findings suggest that NR1D1 deficiency impairs macrophage‐mediated efferocytosis, thereby contributing to unresolved inflammation in colitis.

### 2.3 HA@Alg‐ADAP‐SR9011 Enables Sustained NR1D1 Activation and Ameliorates Intestinal Inflammation

To test the therapeutic potential of NR1D1 activation in colitis, we administered the synthetic agonist SR9011 to mice subjected to DSS‐induced inflammation. Systemic delivery achieved limited intestinal exposure, prompting us to pursue a localized strategy. To overcome this limitation while aligning exposure with circadian dynamics, we sought a localized delivery platform capable of sustained, mucosa‐adherent release in the colon.

For hydrogel design, we used Andrias davidianus—a long‐lived amphibian with exceptional regenerative and hypoxia‐tolerant traits thought to derive from bioactive peptides. Peptides extracted from skin and muscle (ADAP, A. davidianus active peptides) were sequenced by mass spectrometry and screened for SR9011 interaction by molecular docking (Figure S7, Supporting Information), which revealed a stable complex, suggesting delayed release. SR9011 was first adsorbed onto ADAP, blended into sodium alginate, and emulsified via microfluidic flow‐focusing to yield monodisperse microspheres. These were cross‐linked in CaCl_2_ and subsequently coated with 1% hyaluronic acid (HA) to enhance mucosal adhesion. The resulting HA@Alg‐ADAP‐SR9011 microspheres provided a formulation for localized and sustained drug delivery to the inflamed colon.

**Figure 4:**
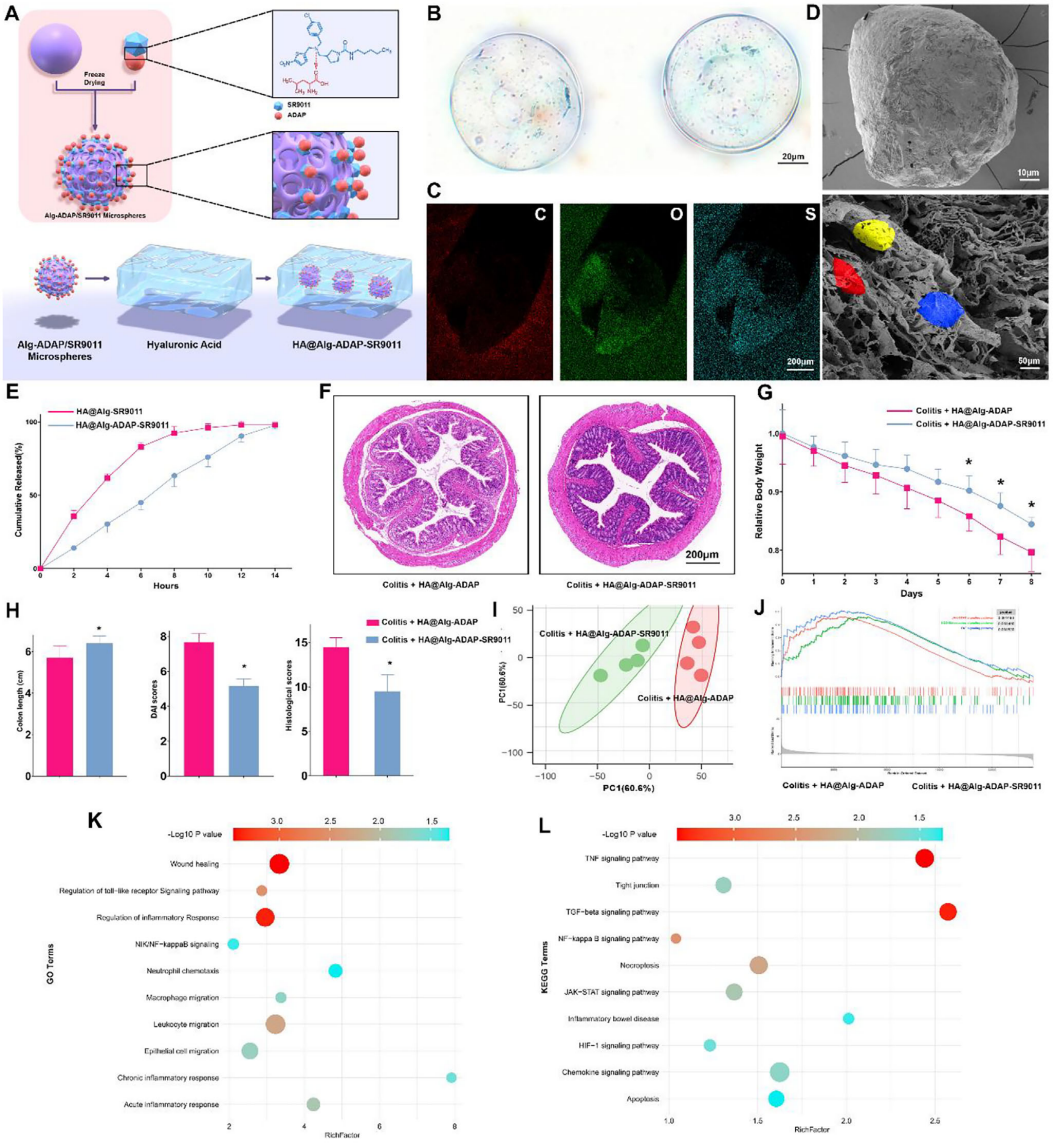
HA@Alg‐ADAP‐SR9011 hydrogel enables sustained SR9011 delivery and ameliorates DSS‐induced colitis. A) Schematic workflow for preparation of HA@Alg‐ADAP‐SR9011 microspheres. B) Optical microscopy showing uniform microsphere morphology and size distribution (*n* = 6). C) Energy‐dispersive spectroscopy (EDS) elemental mapping of C, O, and S demonstrating homogeneous ADAP incorporation (*n* = 6). D) Scanning electron microscopy (SEM) of microspheres before and after HA coating, visualizing core–shell architecture (*n* = 6). E) Cumulative SR9011 release from HA@Alg‐SR9011 versus HA@Alg‐ADAP‐SR9011 (*n* = 6). F) Representative H&E‐stained colon sections from DSS‐treated mice receiving HA@Alg‐ADAP or HA@Alg‐ADAP‐SR9011 (*n* = 6). G) Body‐weight trajectories during DSS treatment, showing reduced weight loss in HA@Alg‐ADAP‐SR9011 mice (mean ± S.D., unpaired t‐test, *n* = 6, ^*^ *p* <0.05). H) Colon length, DAI, and histological scores, all improved in HA@Alg‐ADAP‐SR9011 mice (mean ± S.D., unpaired t‐test, *n* = 6, ^*^ *p* <0.05). I) PCA of bulk RNA‐seq from IECs, demonstrating distinct clustering of SR9011‐treated versus control groups (*n* = 4). J) GSEA showing suppression of TNFα signaling and other inflammatory pathways in SR9011‐treated mice (*n* = 4). K) GO enrichment analysis of differentially expressed genes, highlighting reduced inflammatory response and leukocyte migration (*n* = 4). L) KEGG pathway analysis confirming downregulation of UC–associated signaling (*n* = 4).

**Figure** **4**A shows the schematic workflow for preparing HA@Alg‐ADAP‐SR9011. Optical microscopy confirmed uniform microsphere morphology and size distribution (Figure 4B). Energy dispersive spectroscopy and elemental mapping demonstrated a homogeneous sulfur signal, indicating even dispersion of ADAP peptides within the microspheres (Figure 4C). Scanning electron microscopy further revealed a stable core–shell architecture before and after HA coating (Figure 4D). Release assays showed that ADAP incorporation markedly delayed SR9011 release, with ≈80% released over 10 h (Figure 4E).

We next assessed the physicochemical and functional properties of the hydrogel. Incorporation of ADAP significantly increased drug loading (DL%) and encapsulation efficiency (EE%), indicating enhanced stabilization of SR9011 within the matrix (Figure S8A,B, Supporting Information). Laser diffraction confirmed monodisperse microspheres with narrow D10/D50/D90 distributions, and ζ‐potential measurements across pH 2.0, 6.0, and 7.4 showed a shift toward more negative charges after HA coating, consistent with improved mucoadhesion (Figure S8C,D, Supporting Information). Atomic force microscopy further demonstrated a uniform nanoscale HA layer (Figure S8E, Supporting Information).

In vitro release assays under sequential gastric, intestinal, and colonic conditions showed sustained SR9011 release from HA@Alg‐ADAP‐SR9011 compared with HA@Alg‐SR9011, with mass‐balance analyses confirming near‐complete recovery (Figure S8F, Supporting Information). Mucoadhesion tests under physiologically relevant shear revealed stronger and more persistent adhesion for HA‐coated microspheres than uncoated controls (Figure S8G, Supporting Information). In vivo, DiR‐labeled formulations demonstrated uptake of SR9011 by colonic epithelial cells at 0, 6, and 12 h, confirming targeted delivery and retention (Figure S8H, Supporting Information).

To incorporate circadian context, we adopted a Zeitgeber‐time (ZT) framework in both experimental design and data presentation, enabling direct comparison of in‐phase and out‐of‐phase dosing under matched exposure. Pharmacokinetic profiling of colonic tissue by validated LC–MS/MS showed that ADAP incorporation extended SR9011 exposure, reduced peak–trough variability, and improved tissue retention compared with alginate‐only microspheres (Figure S9A,B, Supporting Information). We did not measure SR9011 concentrations in plasma or other organs; thus, our conclusions are based on preliminary indications from colonic tissue data and IEC/LP fractionation.

We next examined circadian oscillations of NR1D1, BMAL1, and CD47. Under in‐phase dosing, HA@Alg‐ADAP‐SR9011 preserved rhythmic repression of *Cd47*, whereas mismatched dosing dampened these oscillations (Figure S9C–G, Supporting Information). SR9011 did not change *Nr1d1* expression but significantly suppressed Bmal1 and *Cd47* in IECs, with no detectable effect in the lamina propria (spatial omics analyses were not performed), indicating that its activity may be mainly confined to the epithelium (Figure S9H,I, Supporting Information). Western blotting of IECs at ZT6 confirmed reduced BMAL1 and CD47 protein levels (Figure S9J,K, Supporting Information). By contrast, rectal administration of free SR9011 altered BMAL1 and CD47 expression only transiently, at a single post‐treatment time point (Figure S9L,M, Supporting Information).

To assess the biocompatibility and safety of the hydrogel system, we expanded both in vitro and in vivo analyses. In colonic organoids, exposure to ADAP, SR9011, or HA@Alg‐ADAP‐SR9011 for 48 h did not increase reactive oxygen species (ROS) generation, as measured by flow cytometry, nor did it impair cell viability, as determined by CCK‐8 assays (Figure S10A,B, Supporting Information). These findings indicate that the formulations do not induce oxidative stress or cytotoxicity. For in vivo safety assessment, mice were administered HA@Alg‐ADAP daily for 8 days. No differences were observed in body weight, behavior, colon length, or histopathological features compared with controls (Figure S10C,D, Supporting Information). Together, these results demonstrate that the hydrogel carrier is well tolerated and biocompatible in vivo.

In DSS‐induced colitis, concomitant treatment with HA@Alg‐ADAP‐SR9011 ameliorated both clinical and histological disease features. H&E staining revealed reduced mucosal damage (Figure 4F), while treatment mitigated body weight loss, preserved colon length, and significantly lowered histological inflammation scores and DAI values (Figure 4G,H). Bulk RNA‐seq of IECs showed distinct transcriptional profiles between HA@Alg‐ADAP‐SR9011–treated and HA@Alg‐ADAP controls, as indicated by PCA (Figure 4I). GSEA demonstrated marked suppression of pro‐inflammatory pathways, including TNFα signaling (Figure 4J). GO and KEGG enrichment further highlighted broad modulation of inflammation‐related pathways by SR9011 (Figure 4K,L).

Together, these results demonstrate that HA@Alg‐ADAP‐SR9011 provides sustained, intestine‐targeted NR1D1 activation, attenuating inflammation and promoting mucosal homeostasis in experimental colitis.

### 2.4 SR9011 Enhances Efferocytosis and Restores Intestinal Homeostasis in Colitis

**Figure 5:**
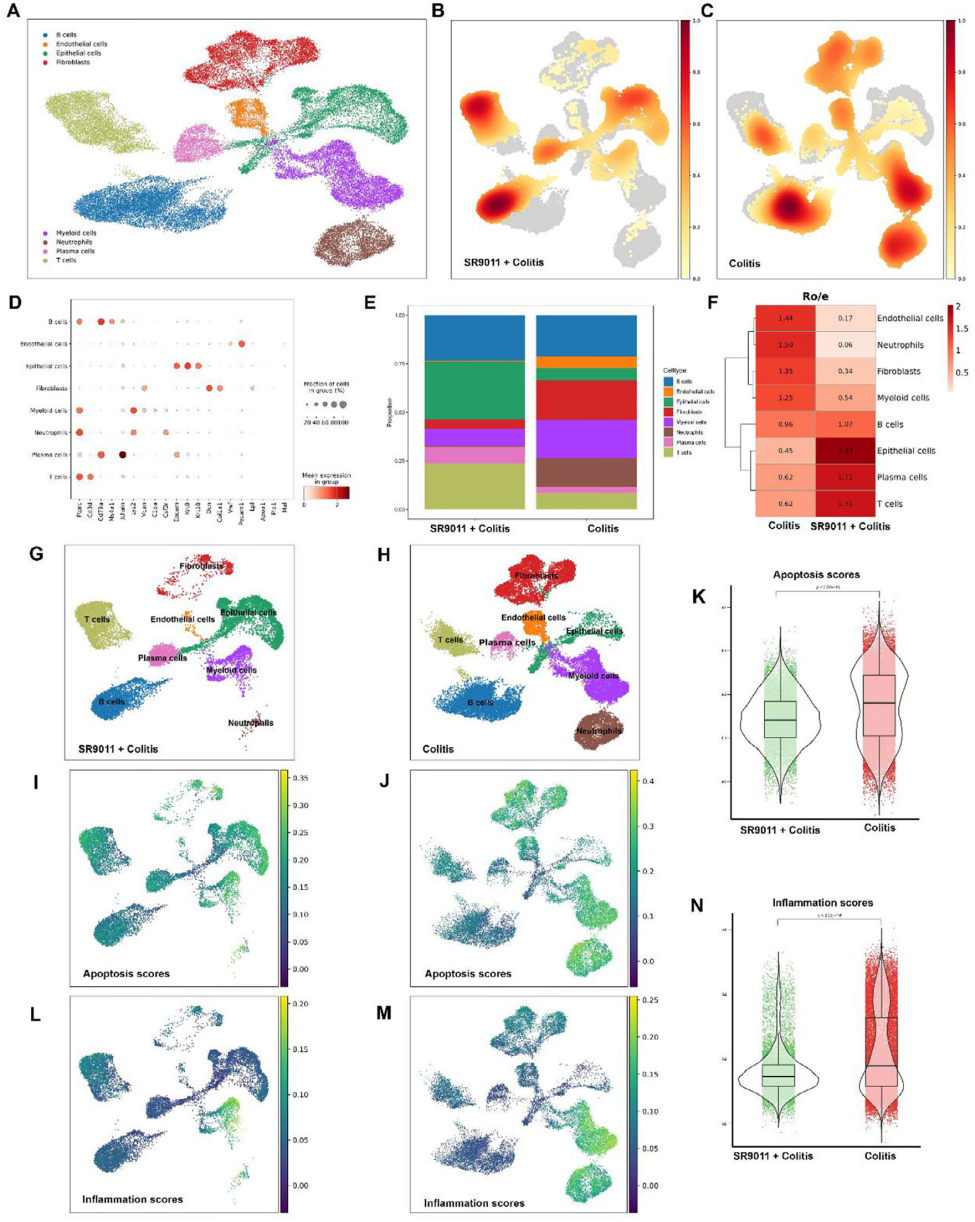
Single‐cell transcriptomic profiling reveals SR9011 reduces apoptosis and inflammation in colonic cell populations during colitis. A) UMAP projection of scRNA‐seq from colonic tissues of DSS‐treated mice with or without SR9011, identifying epithelial, fibroblast, endothelial, T, B, plasma, myeloid, and neutrophil populations (*n* = 4 per group). B,C) Density plots showing cell‐type abundance, with SR9011 treatment increasing epithelial cells and reducing fibroblasts and neutrophils (*n* = 4). D) Dot plot of canonical marker expression for cell‐type annotation; dot size reflects the fraction of expressing cells, and color intensity indicates mean expression (*n* = 4). E) Bar plot of relative cell‐type proportions, highlighting expansion of epithelial cells and depletion of fibroblasts and neutrophils in SR9011‐treated mice (*n* = 4). F) Heatmap of enrichment ratios (Ro/e) demonstrating compositional shifts after SR9011 treatment (*n* = 4). G,H) UMAP clustering of treatment and control groups showing distinct distributional changes. J) UMAP mapping of apoptosis scores in SR9011‐treated versus untreated mice, with reduced apoptosis after treatment (*n* = 4). K) Violin plot comparing apoptosis scores between groups, showing significantly lower values with SR9011 (unpaired t‐test, ^*^ *p* <0.01). L,M) UMAP mapping of inflammation scores across groups, indicating suppression of inflammatory signaling with SR9011. N) Violin plot quantifying inflammation scores, confirming significant reductions following treatment (unpaired t‐test, *P < 0.01).

To dissect the cellular basis of SR9011's protective effects, we performed single‐cell RNA sequencing (scRNA‐seq) on colonic tissues from DSS‐induced colitis mice with or without SR9011 treatment. Single‐cell suspensions were processed on the 10x Genomics Chromium platform, yielding 40943 high‐quality cells from an initial 60695 across eight biological replicates. Scanpy analysis identified major cell lineages, including epithelial cells, fibroblasts, and immune subsets such as T cells, B cells, neutrophils, and plasma cells (**Figure** **5**A).

SR9011 treatment shifted cellular composition, with expansion of epithelial cells and depletion of fibroblasts and neutrophils (Figure 5B–E). These changes were confirmed by enrichment heatmaps and UMAP projections (Figure 5F–H). Functional scoring revealed broadly reduced apoptosis and inflammation signatures across multiple cell types in the SR9011 group (Figure 5I–N), indicating suppression of pro‐apoptotic and pro‐inflammatory programs in the colonic microenvironment.

Given the pronounced reduction in fibroblasts, we hypothesized that epithelial–mesenchymal transition (EMT) may drive fibroblast accumulation during inflammation. Joint analysis of epithelial and fibroblast populations could thus clarify how SR9011 modulates EMT and its impact on CD47 regulation.

**Figure 6:**
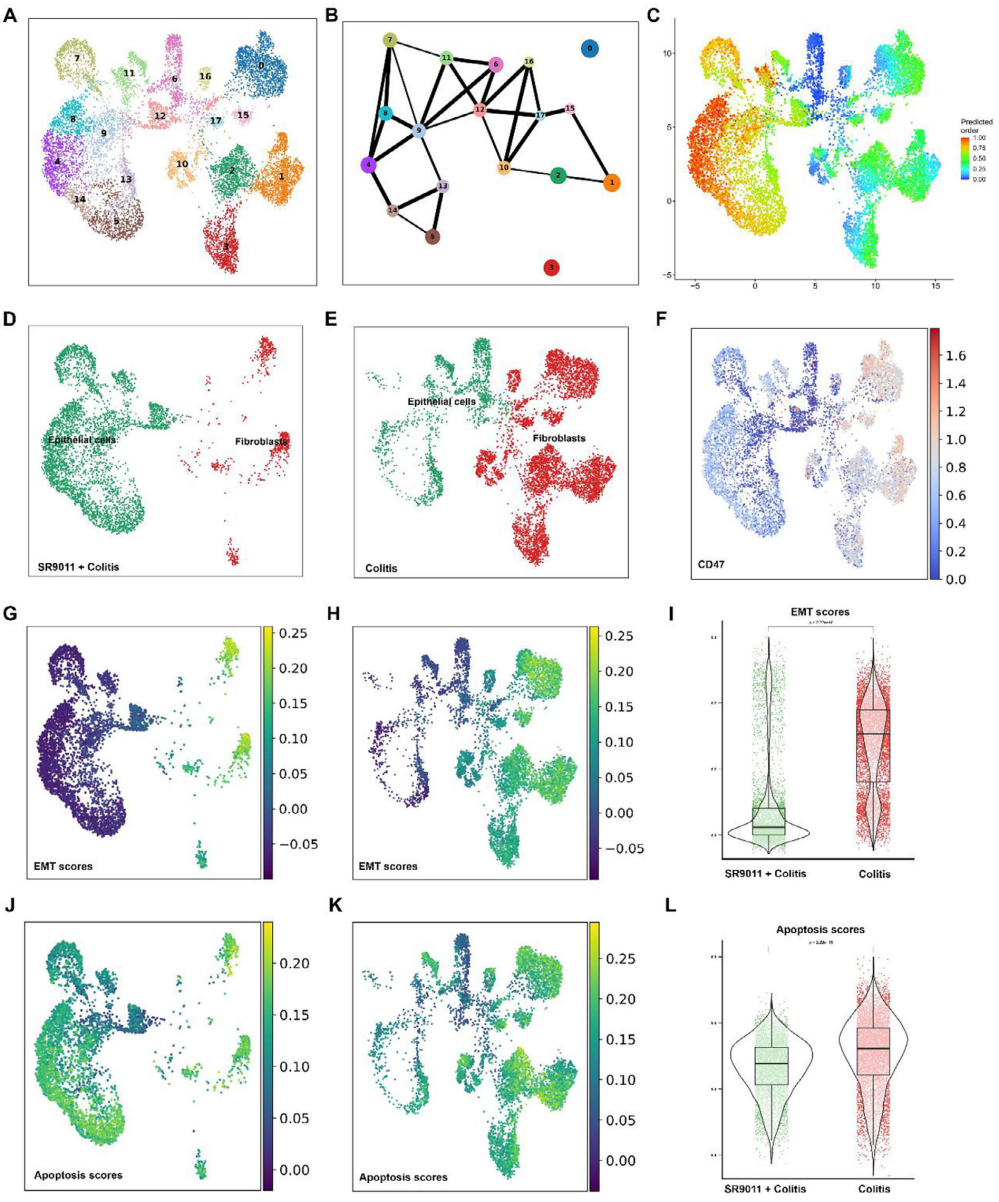
SR9011 reduces CD47 expression, suppresses EMT, and limits apoptosis in epithelial and fibroblast populations. A) UMAP plot showing subclustering of epithelial and fibroblast populations into 18 transcriptionally distinct subsets (*n* = 4). B) Partition‐based graph abstraction (PAGA) illustrating connectivity among subclusters, indicating lineage continuity (*n* = 4). C) Cytotrace analysis projecting stemness potential, with higher scores on the left reflecting progenitor‐like states (*n* = 4). D,E) UMAP plots of epithelial and fibroblast distributions in SR9011‐treated (D) and untreated (E) colitis mice, showing reduced fibroblast abundance after treatment (*n* = 4). F) UMAP heatmap of CD47 expression across epithelial and fibroblast clusters, demonstrating reduced expression with SR9011 (*n* = 4). G,H) EMT score mapping in SR9011‐treated (G) and untreated (H) mice (*n* = 4). I) Violin plot quantifying EMT scores, confirming significant suppression of EMT by SR9011 (unpaired t‐test, ^*^ *p* <0.01). J,K) Apoptosis score mapping in SR9011‐treated (J) and untreated (K) groups, showing reduced apoptosis after treatment. L) Violin plot comparing apoptosis scores between groups, demonstrating significant reduction with SR9011 (unpaired t‐test, ^*^ *p* <0.01).

To further investigate EMT dynamics, we subclustered epithelial and fibroblast populations, identifying 18 transcriptionally distinct subsets (**Figure** **6**A). Partition‐based Graph Abstraction (PAGA) revealed strong interconnectivity, consistent with lineage continuity (Figure 6B). Cytotrace analysis showed a stemness gradient, with less differentiated populations positioned to the left of the trajectory (Figure 6C). UMAP projections highlighted distinct distributions between SR9011‐ and vehicle‐treated groups (Figure 6D,E).

Within this epithelial–fibroblast continuum, SR9011 treatment reduced CD47 expression (Figure 6F), suppressed EMT scores (Figure 6G–I), and lowered apoptosis across epithelial and fibroblast clusters (Figure 6J–L). These findings support a model in which NR1D1 activation restrains EMT, limits apoptosis, and enhances efferocytosis, thereby promoting resolution of colonic inflammation.

**Figure 7:**
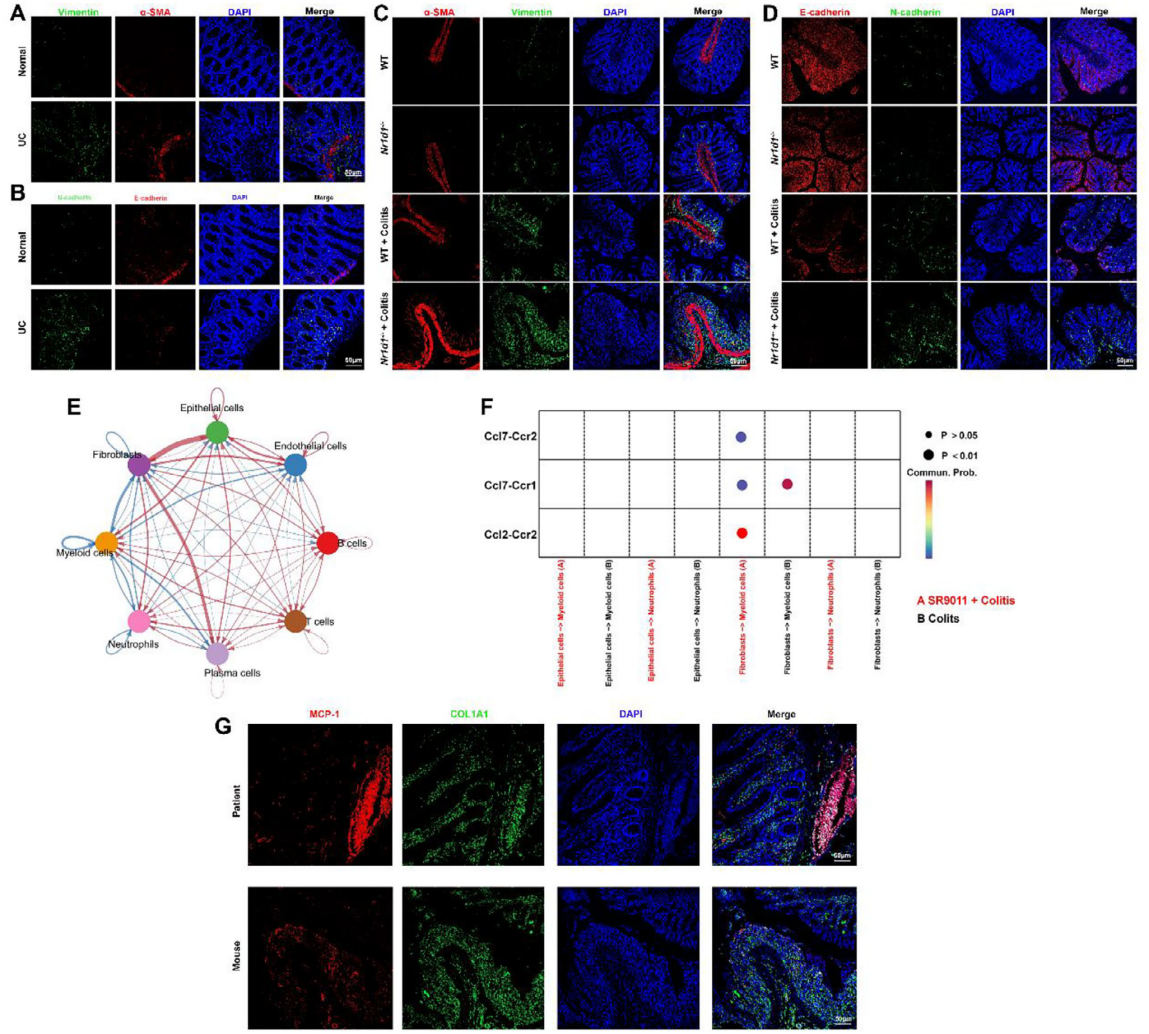
In vivo validation of EMT activation and fibroblast–macrophage crosstalk in UC patients and colitis mice. A,B) Immunofluorescence of colonic mucosa from healthy controls and UC patients showing increased mesenchymal markers α‐SMA, Vimentin (A), and N‐cadherin (B), with reduced epithelial marker E‐cadherin in UC (*n* = 6). C,D) Colonic tissues from WT and *Nr1d1^−/−^* mice with or without DSS‐induced colitis, showing that NR1D1 deficiency exacerbates EMT, with elevated α‐SMA, Vimentin, and N‐cadherin (C) and reduced E‐cadherin (D) (*n* = 6). E) Cell–cell communication network from scRNA‐seq, showing strong interaction potential among epithelial cells, fibroblasts, and macrophages in colitis (*n* = 4 per group). F) Ligand–receptor heatmap highlighting fibroblast‐derived CCL2 signaling to macrophage CCR2. Dot size indicates communication probability; color denotes group origin. G) Co‐immunofluorescence for CCL2 (red) and COL1A1 (green) in colonic tissues from IBD patients and DSS mice confirms fibroblast‐derived CCL2 expression in situ (*n* = 6).

Because scRNA‐seq provides inferential rather than definitive evidence of EMT, we validated these observations in vivo. Immunofluorescence staining of colonic biopsies from UC patients and DSS‐treated mice demonstrated elevated EMT markers (α‐SMA, Vimentin, N‐cadherin) and reduced E‐cadherin, confirming EMT activation (**Figure** **7**A–D).

Finally, to explore fibroblast–macrophage interactions, we conducted ligand–receptor analysis, which revealed robust signaling among epithelial cells, fibroblasts, and macrophages (Figure 7E). Fibroblast‐derived CCL2 was predicted to engage macrophage CCR2, a pathway validated by dual immunofluorescence showing colocalization of Collagen I and CCL2 in colonic tissues from IBD patients and DSS mice (Figure 7F,G). These results highlight a fibroblast‐to‐macrophage communication axis that may further shape the inflammatory niche.

## 3 Discussion

Under physiological conditions, macrophages play a key role in resolving inflammation by efficiently clearing apoptotic cells, a process known as efferocytosis. In UC, loss of NR1D1 leads to upregulation of CD47, thereby impairing apoptotic clearance. The accumulation of uncleared apoptotic debris amplifies mucosal inflammation, as defective efferocytosis fails to terminate the inflammatory response, resulting in a vicious cycle of tissue injury.^[^ ^37^, ^38^, ^39^ ^]^ Our in vitro and in vivo experiments provide direct evidence that NR1D1 deficiency promotes apoptotic cell accumulation and exacerbates inflammation through this pathway.

Previous studies have established NR1D1 as a circadian regulator of metabolic, inflammatory, and autophagic processes.^[^ ^40^, ^41^, ^42^ ^]^ Here, we extend its role to the regulation of apoptotic clearance in the gut. For the first time, we demonstrate that modulating the NR1D1–CD47 axis can restore macrophage efferocytic function and mitigate intestinal inflammation. Specifically, we show that SR9011‐activated NR1D1 reduces CD47 expression, enhances macrophage‐mediated clearance of apoptotic cells, and significantly improves disease outcomes in a murine colitis model. These findings suggest that targeting NR1D1–CD47 interactions represents a novel and effective therapeutic strategy for UC.

Our results are consistent with emerging evidence highlighting the importance of impaired efferocytosis in the pathogenesis of chronic inflammatory diseases. For example, Szabo et al. reported that alcohol impairs hepatic macrophage clearance of neutrophil extracellular traps in alcoholic liver disease, exacerbating hepatic inflammation.^[^ ^43^ ^]^ Similarly, Ramirez‐Ortiz et al. showed that impaired macrophage efferocytosis in lupus leads to increased Tfh2 cell responses and autoantibody production.^[^ ^44^ ^]^ Wu et al. further demonstrated that enhancing macrophage efferocytosis alleviates intestinal inflammation, while Morioka et al. emphasized that timely apoptotic cell clearance is crucial for controlling inflammation.^[^ ^45^, ^46^ ^]^ These studies collectively support our findings that improving macrophage efferocytosis can significantly mitigate inflammatory responses and promote tissue repair.

To achieve sustained intestinal delivery of SR9011 and better mimic the physiological oscillation of NR1D1, we developed a bioactive hydrogel system based on Andrias davidianus active peptides (ADAP). Compared to conventional hydrogels, peptide‐based hydrogels offer superior biocompatibility, lower immunogenicity, and customizable functionalization.^[^ ^47^, ^48^ ^]^ The HA@Alg‐ADAP‐SR9011 hydrogel exhibited prolonged SR9011 release, closely aligning with the circadian expression rhythm of NR1D1. This design not only enhanced the therapeutic efficacy but also minimized potential off‐target effects. We note, however, that SR9011 concentrations were not assessed in plasma or extraintestinal organs, and spatial omics analyses were not performed. Preliminary evidence of localized retention is instead supported by colonic pharmacokinetics and IEC/LP fractionation.

Despite encouraging results, circadian‐based drug therapy faces key hurdles: patient‐specific variability in circadian rhythms driven by genetics, environment, and lifestyle; potential misalignment between disease activity cycles and clock gene oscillations; and the translational challenges of scaling and regulating peptide‐based hydrogels. Moreover, SR9011 remains experimental, underscoring the need for safer, clinically optimized NR1D1 agonists. Nonetheless, integrating circadian pharmacology with targeted biomaterial delivery offers strong translational promise. By aligning drug exposure with rhythmic biology, such systems may enhance efficacy, reduce systemic toxicity, and restore temporal homeostasis. In UC, where inflammation and repair fluctuate diurnally, circadian‐aligned therapies could provide distinct advantages, warranting biomarker‐guided dosing strategies, improved NR1D1 modulators, and long‐term safety studies.

In conclusion, our findings establish NR1D1 as a key transcriptional regulator linking epithelial apoptosis, efferocytosis, and intestinal inflammation. Targeted activation of NR1D1 through rhythm‐sensitive hydrogel‐based delivery of SR9011 restores mucosal immune balance and attenuates colitis. These results provide a compelling rationale for the development of NR1D1‐based chronotherapeutic strategies for the treatment of UC and other chronic inflammatory diseases.

## 4 Conclusion

We identify NR1D1 as a key transcriptional repressor of CD47 that coordinates epithelial apoptosis and macrophage‐mediated efferocytosis in UC. NR1D1 deficiency impairs apoptotic clearance, exacerbates mucosal inflammation, and promotes epithelial dysfunction. Pharmacological activation of NR1D1 using a bioadhesive hydrogel delivering SR9011 restores intestinal immune balance, reduces CD47 expression, and ameliorates colitis severity. These findings uncover a critical NR1D1–CD47 regulatory axis controlling intestinal homeostasis and highlight rhythm‐sensitive NR1D1 activation as a promising therapeutic strategy for UC.

## 5 Experimental Section

### Key Reagents and Antibodies

For Western blotting, antibodies against NR1D1 (Cat# 13418S) and GAPDH (Cat# 5174S) were from Cell Signaling Technology (Danvers, MA, USA) human CD47 (Cat# ab284132), and mouse CD47 (Cat# ab214453) were from Abcam (Cambridge, UK). ChIP assays used anti‐NR1D1 (Cat# 13418S) and anti‐rabbit IgG (Cat# 14708S, Cell Signaling Technology). For immunofluorescence, NR1D1 (Cat# 13418S) and COL1A1 (Cat# 66948) were from Cell Signaling Technology; E‐Cadherin, α‐SMA, Vimentin, N‐Cadherin, ZO1 (Cat# ab221547), Occludin (Cat# ab216327), and MCP‐1 (Cat# ab214819) were from Abcam. For flow cytometry, Annexin V‐FITC/7‐AAD (Cat# 40311ES), CMTPX (Cat# 4017ES), and CMFDA (Cat# 40721ES) were obtained from Yeasen Biotechnology (Shanghai, China). Chemicals included LPS (Cat# 497693, Thermo Fisher Scientific, Waltham, MA, USA), staurosporine (Cat# S5921, Sigma‐Aldrich, St. Louis, MO, USA), shRNA and luciferase reporter plasmids (Qijing Biological Technology, Wuhan, China), iMAX transfection reagent (Cat# 13778150, Thermo Fisher Scientific), DSS (Cat# 9011‐18‐1, MP Biomedicals, Solon, OH, USA), and SR9011 (Cat# SML2067, Sigma‐Aldrich, St. Louis, MO, USA).

### Patient Tissue Sampling

Tissue samples were collected from 324 individuals at Wuhan Union Hospital between September 2020 and October 2022, comprising 219 patients with active UC and 105 controls with non‐inflammatory, non‐neoplastic conditions undergoing colonoscopy. UC diagnosis was based on clinical symptoms and Mayo endoscopic subscore (MES), with 107 patients classified as MES 1 and 112 as MES 2–3. At six‐month follow‐up, 126 patients in remission who completed repeat colonoscopies were further assessed. Biopsies from UC patients were obtained from inflamed rectal regions and matched to control sites. Each specimen was divided for paraformaldehyde fixation (immunofluorescence) or preservation for qPCR, Western blotting, and CUT&Tag; samples for qPCR were snap‐frozen and stored at −80 °C. All participants provided informed consent, and the study was approved by the Independent Ethics Committee of Wuhan Union Hospital. Demographic and clinical characteristics are summarized in Table S1 (Supporting Information).

### Crypt Isolation, Organoid Culture, and shRNA Knockdown

Crypt isolation and organoid culture were performed as described previously.^[^ ^35^ ^]^ Non‐tumorous colonic tissue from colorectal cancer patients confirmed cancer‐free by pathology was cut longitudinally and rinsed with cold PBS. Crypts were released by incubation with Gentle Cell Dissociation Reagent (STEMCELL Technologies, Seattle, WA, USA) on a rocking platform (20 rpm) for 20 min at room temperature and passed through a 70‐µm strainer, repeated four times. The crypt fraction was enriched by centrifugation (290 g, 5 min, and 4 °C), resuspended in 0.1% BSA/PBS, transferred to a fresh tube, and pelleted twice at 200 g for 3 min in cold DMEM/F‐12 with 15 mM HEPES (STEMCELL Technologies). Final crypt pellets were embedded in Matrigel (BD Biosciences, San Jose, CA, USA), plated in 24‐well plates, and overlaid with 750 µL pre‐warmed IntestiCult Organoid Growth Medium (STEMCELL Technologies, Vancouver, Canada). Medium was refreshed every 2 days to sustain organoid growth. For gene silencing, organoids were transfected with shRNA constructs targeting NR1D1 (sequence: CCAGCCCTGAATCCCTCTATA) or CD47 (sequence: GCCTTGGTTTAATTGTGACTT) using standard protocols.

### Animal Models and Experimental Design

Wild‐type C57BL/6J mice were purchased from Cavens (Changzhou, China). Intestinal epithelial cell‐specific *Nr1d1* knockout mice (*Nr1d1* ^−/−^) were generated by crossing *Nr1d1* ^flox/flox^ mice (Cyagen, Guangzhou, China) with Villin‐Cre mice, both on a C57BL/6J background and engineered using CRISPR/Cas9. Cohorts comprised six mice per group, with no data excluded. Mice were housed under controlled conditions with free access to chow and water. Acute colitis was induced with 2.5% (w/v) DSS administered in drinking water for 8 days, after which animals were euthanized and colonic tissues collected. A 0.5‐cm distal segment was reserved for histology (H&E, immunofluorescence), a 2‐cm segment for single‐cell sequencing, and the remainder for IEC isolation followed by proteomics, transcriptomics, Western blotting, and qPCR. The primary endpoint was disease severity, assessed by body weight change, colon length, and histopathological score at day 8; secondary endpoints included molecular analyses in IECs. All procedures complied with institutional ethical guidelines (Tongji Medical College, Huazhong University of Science and Technology).

### Preparation of ADAP‐Based Hydrogel Microspheres for SR9011 Delivery (HA@Alg‐ADAP‐SR9011)

Sodium alginate and hyaluronic acid (HA; 100–300 kDa) were obtained from Sigma‐Aldrich (St. Louis, MO, USA). Andrias davidianus active peptides (ADAP) were extracted, lyophilized (Hbyounike, Wuhan, China), and sequenced by mass spectrometry (Sangon Biotech, Shanghai, China). ADAP was dissolved in PBS (50 mg mL^−1^), and SR9011, pre‐dissolved in DMSO (<0.1% v/v), was diluted to 10 mg mL^−1^ and incubated with ADAP for 2 h to allow adsorption. The ADAP–SR9011 complex was mixed with 2% (w/v) sodium alginate, degassed, and emulsified using a flow‐focusing microfluidic device, with aqueous and oil phases infused at 10–20 and 100–150 µL min^−1^, respectively. Monodisperse droplets (≈50–100 µm) were cross‐linked in 2% CaCl_2_ for 15 min, harvested by centrifugation (1000 rpm, 5 min), and washed with PBS. For HA‐coating, microspheres were incubated in 1% HA for 30 min, yielding HA‐coated alginate/ADAP/SR9011 microspheres—hereafter referred to as HA@Alg‐ADAP‐SR9011—of ≈200–300 µm, which were collected by mild centrifugation (500 rpm, 5 min) and washed before use. During DSS treatment, mice received a 100 µL suspension of HA@Alg‐ADAP‐SR9011 once daily by oral gavage.

### Mass Spectrometry Detection and Docking Analysis of ADAP

Andrias davidianus peptides were dissolved in 0.1% formic acid with 2% acetonitrile, filtered through a 10 kDa cutoff device (12000 × g, 10 min), desalted on a C18 column, eluted with 60% acetonitrile/0.1% formic acid, dried, and reconstituted in 0.1% formic acid. Peptides were analyzed on an Easy‐nLC 1200 coupled to a Q Exactive mass spectrometer (Thermo Fisher Scientific) using a C18 column (75 µm × 25 cm, 2 µm, 100 Å) with a 30‐min 5–38% acetonitrile gradient at 300 nL min^−1^. Spectra were acquired in data‐dependent mode with full MS scans at 70000 resolution and MS/MS at 28 eV. For docking analysis, the Schrödinger software suite was employed to model the interaction between ADAP peptides and SR9011.

### Physicochemical Characterization and Functional Evaluation of Microspheres

Drug loading (DL%) and encapsulation efficiency (EE%) were determined by a standard mass‐balance method to evaluate the actual quantitative proportion of SR9011 successfully entrapped within the hydrogel matrix. The amount of drug incorporated into microspheres was calculated by subtracting the quantity of SR9011 detected in the supernatants and wash fractions from the total amount initially added during formulation. The dry mass of microspheres was obtained after lyophilization to constant weight, ensuring accurate normalization of drug content. Based on these measurements, the following formulas were applied. DL% was defined as (𝑚_drug, in beads_/_𝑚beads, dry_) × 100(_mdrug, in beads_/m_beads, dry_) × 100, and EE% as (𝑚_drug, in beads_/𝑚_drug, feed_) × 100(mdrug, in beads/mdrug, feed) × 100.

Particle size distributions (D10, D50, D90) of alginate–ADAP–SR9011 droplets and HA‐coated microspheres were measured using a laser diffraction particle size analyzer (Accusizer 780, Particle Sizing Systems, USA). HA layer thickness was determined by atomic force microscopy (Dimension ICON, Bruker, USA) in tapping mode, with cross‐sectional height profiles extracted from immobilized coated and uncoated microspheres to calculate the mean increment attributable to HA.

Surface charge was assessed using a Zetasizer Nano ZS90 (Malvern Panalytical, UK) with a DTS1070 folded capillary cell at 25 °C. Uncoated and HA‐coated microspheres (≈0.05–0.10% w/v) were dispersed in electrolyte‐adjusted buffers at pH 2.0 (0.01 M HCl/NaCl), pH 6.0 (10 mM MES/NaCl), or pH 7.4 (10 mm PBS/NaCl), equilibrated for 5 min, and gently inverted to avoid bubbles (pre‐cleared at 500 g, 2 min). Measurements were acquired in automatic voltage mode with backscatter detection (173°), and ζ‐potentials were derived from electrophoretic mobility using the Smoluchowski model (f(κa) = 1.5).

In‐vitro release of SR9011 from HA@Alg‐SR9011 and HA@Alg‐ADAP‐SR9011 microspheres was assessed sequentially in simulated gastric (SGF, pH 1.2, 0–2 h), intestinal (SIF, pH 6.8, 2–6 h), and colonic fluids (SCF, pH 7.4, 6–14 h) at 37 °C with gentle agitation. At specified intervals, supernatants were collected, centrifuged, and analyzed for SR9011 content by LC–MS/MS, with fresh medium replenished to maintain sink conditions. Based on these measurements, mass balance was determined as the percentage of SR9011 released, retained, or lost, with results reported as mean ± s.d. from at least three independent batches.

Mucoadhesion was evaluated under physiologically relevant shear using excised porcine esophageal mucosa maintained at 37 °C with native mucus preserved. Tissue was mounted in a custom flow chamber perfused with simulated saliva at a defined volumetric flow rate generating a shear stress of X Pa (calculated from channel geometry). Hydrogel samples were applied to the mucosal surface, and residual coverage was quantified by image analysis and mass recovery at 0.5, 1, 2, 6, and 14 h.

### Quantification of SR9011 in Mouse Colon by LC–MS/MS

Colon concentrations of SR9011 were determined by a validated LC–MS/MS assay (Sangon Biotech, Shanghai). Colons collected after administration of HA@Alg‐ADAP‐SR9011 or HA@Alg‐SR9011 were weighed, homogenized (1:4, w/v) in 50:50 H_2_O:acetonitrile with 0.1% formic acid, and extracted by acetonitrile precipitation with an isotopically labelled internal standard. Supernatants were dried, reconstituted, and analyzed on a C18 column with a water/acetonitrile (0.1% formic acid) gradient (0.4 mL min^−1^). Detection was performed in positive‐ion electrospray MRM with optimized transitions, providing ≥12 points per peak. Calibration was matrix‐matched (blank colon homogenate) over 1–5000 ng·g^−1^ with a low‐ng g^−1^ LLOQ and weighted (1/x^2^) regression. Accuracy, precision, recovery, stability, and matrix effects met bioanalytical criteria.

### Visualization of SR9011 Uptake In Mouse Colonic Tissue

To track intestinal uptake of SR9011, the drug was labeled with the lipophilic fluorescent dye DiR (Thermo Fisher Scientific, Waltham, MA, USA) and incorporated into the HA@Alg‐ADAP‐SR9011 formulation. Mice received DiR‐tagged microspheres and were euthanized at 0, 6, or 12 h. Colons were harvested, sectioned, and examined by fluorescence microscopy to detect near‐infrared DiR signals within colonic tissue.

### Isolation of Intestinal Epithelial Cells (IECs)

Colon samples were rinsed with ice‐cold DPBS, cut into 0.5‐cm fragments, and incubated in EDTA/DTT buffer (14 ml DPBS, 0.9 ml 0.5 M EDTA, 22.5 µL 1 M DTT) for 75 min at 4 °C to dissociate the epithelial layer, as described previously.^[^ ^36^ ^]^ The tissue fragments were then subjected to three sequential rounds of centrifugation and resuspension in fresh EDTA/DTT buffer to maximize IEC yield. Supernatants containing released IECs were pooled, filtered through a 40‐µm mesh, and used for downstream molecular analyses, including qPCR, Western blotting, proteomics, and bulk RNA sequencing. The epithelial‐depleted tissue was retained as a lamina propria–enriched fraction (LP fraction) for complementary analyses.

### Human Monocyte‐Derived Macrophages (THP‐1 Cells)

In this study, human THP‐1 acute monocytic leukemia cells were utilized to derive macrophages. THP‐1 cells were cultured in RPMI‐1640 medium (Gibco, Waltham, MA, USA), supplemented with 10% heat‐inactivated fetal bovine serum (FBS, Gibco, Waltham, MA, USA) and 1% penicillin/streptomycin solution. To induce differentiation into M0 macrophages, the THP‐1 monocytes were treated with 80 ng mL^−1^ phorbol 12‐myristate 13‐acetate (PMA, Sigma, Munich, Germany) for 48 h. After differentiation, the cells were washed and allowed to rest in fresh medium for subsequent experiments.

### Single‐Cell RNA Sequencing and Data Analysis

Single‐cell RNA sequencing (scRNA‐seq) was performed on mouse colonic tissues to investigate the cellular heterogeneity and interactions within the inflamed intestine. Colonic tissues were enzymatically digested with collagenase I and DNase I at 37 °C for 45 min to generate a single‐cell suspension. The resulting suspension was filtered through a 70 µm strainer, followed by red blood cell lysis and additional filtration through a 30 µm strainer. The single‐cell viability was assessed using the Countstar Fluorescence Cell Analyzer, and dead cells were removed using the MACS Dead Cell Removal Kit (Miltenyi Biotec).

Library preparation was carried out using the Chromium Single Cell 3′ Reagent Kit v3.1 (10X Genomics, Pleasanton, CA, USA), followed by sequencing on an Illumina NovaSeq 6000 platform. Data analysis was conducted using the Scanpy package (version 1.9.3) in Python. Cells with fewer than 200 detected genes or more than 20% mitochondrial gene expression were excluded from the analysis. Normalization was performed using UMI counts, and highly variable genes were selected for downstream analyses. Principal component analysis (PCA) was followed by dimensionality reduction techniques, including t‐SNE and UMAP, to visualize cellular clusters. Graph‐based clustering was employed to identify distinct cell populations, and differential gene expression was analyzed using the Wilcoxon rank‐sum test with a log2 fold‐change cutoff of 1 and a *P*‐value threshold of 0.05.

### Proteomic Profiling

Colonic tissues from mice, specifically from three WT + DSS treated and three *Nr1d1* ^−/−^ + DSS treated mice, were subjected to 4D‐DIA proteomic sequencing at Oebiotech (Shanghai, China). Each sample comprised IECs meticulously isolated from the respective mouse models. The advanced mass spectrometric analysis facilitated high‐resolution protein identification and quantification. Data processing, including normalization, differential expression analysis, and visualization, was adeptly performed using Rstudio.

### Bulk RNA Sequencing

IECs were isolated from colonic tissues, and total RNA was extracted with TRIzol (Invitrogen, USA). RNA integrity was verified on an Agilent 2100 Bioanalyzer, and only samples with RIN ≥ 7.0 were used. Poly(A)+ RNA was enriched with oligo(dT) beads, fragmented, and converted to cDNA. Libraries were prepared by end repair, adaptor ligation, and PCR amplification, assessed on a Bioanalyzer, and sequenced on an Illumina NovaSeq 6000 platform (150 bp paired‐end).

Raw reads were trimmed with Trimmomatic and aligned to the mouse genome (mm10) using HISAT2. Gene counts were generated with featureCounts, and differential expression was analyzed with DESeq2 (|log2FC| ≥ 1, FDR < 0.05). Functional enrichment (GO, KEGG, GSEA) and PCA were performed in R using clusterProfiler.

### CUT&Tag Analysis

CUT&Tag (Tn5‐mediated) was performed on mucosal samples from patients with active UC and healthy controls. Nuclei were isolated, immobilized on concanavalin A–coated beads, and incubated with a primary antibody against NR1D1 followed by a secondary antibody. Protein A–Tn5 transposase loaded with sequencing adapters was tethered to antibody–chromatin complexes and activated to cleave and simultaneously tag DNA. Released fragments were extracted, PCR‐amplified, and sequenced at Frasergen (Wuhan, China). Raw reads were quality‐filtered, aligned to the human genome (hg38), and subjected to peak calling to define NR1D1‐binding sites genome‐wide.

### Histological Staining

Sections of the distal colon (0.5 cm) were taken from euthanised mice, fixed in 10% neutral‐buffered formalin at room temperature overnight, and embedded in paraffin. The processed tissue samples were sectioned (5‐µm thick) and subsequently stained with H&E according to conventional procedures, wherein hematoxylin and eosin served as the nuclear stain and the cytoplasmic and extracellular matrix counterstain, respectively. Following this, two independent pathologists, blind to the experimental conditions, conducted a scoring analysis of the sections, evaluating factors such as the degree of inflammation, tissue damage, and infiltration of inflammatory cells. Any disagreements in scoring were resolved through discussion and consensus.

### ELISA Analysis

To quantitatively assess the cytokine milieu within the IEC homogenates, Enzyme‐Linked Immunosorbent Assays (ELISAs) were meticulously executed. The concentrations of key inflammatory mediators, including IL‐10, IL‐1β, TNF‐α, and IL‐17, were measured. Adhering stringently to the manufacturer's instructions, IEC homogenates were defrosted and processed as per the guidelines of the respective ELISA kits. Absorbance readings were obtained at 450 nm using an Enspire microplate reader from PerkinElmer (Waltham, MA, USA). Cytokine levels were deduced from established standard curves, and to ensure precision and consistency, each measurement was performed in triplicate.

### Western Blotting

Proteins were extracted from murine IECs, human colonic IECs, and colonic organoids using RIPA buffer (Thermo Fisher Scientific, MA, USA) supplemented with protease and phosphatase inhibitors (Roche, Basel, Switzerland). Protein concentrations were determined with a BCA Protein Assay Kit (Pierce, IL, USA). Equal amounts (20–30 µg) were separated on 10–12% SDS–PAGE gels and transferred to PVDF membranes (Millipore, MA, USA). Membranes were blocked for 1 h at room temperature in 5% non‐fat milk or BSA in TBS–T, incubated overnight at 4 °C with primary antibodies diluted in TBS–T with 1% BSA, washed, and then incubated with HRP‐conjugated secondary antibodies for 1 h at room temperature. Proteins were detected by enhanced chemiluminescence (GE Healthcare, NJ, USA) and band intensities quantified with ImageJ (NIH, USA), using GAPDH as a loading control.

### Quantitative Real‐Time PCR Analysis

qPCR was performed on a Real‐Time PCR Instrument (Roche Diagnostics, Indiana, USA). Each reaction contained complementary DNA (cDNA), gene‐specific primers, and SYBR Green PCR Master Mix. Thermal cycling conditions were as follows: 95 °C for 3 min, followed by 40 cycles of 95 °C for 15 s, 60 °C for 30 s, and 72 °C for 30 s. Amplification specificity was confirmed by melting curve analysis. All assays were run in triplicate, and relative expression of *CD47* and *NR1D1* was calculated using the 2^–ΔΔCT method with GAPDH as the reference gene.

### Flow Cytometry for Organoid Apoptosis and Efferocytosis

Organoids were treated with lipopolysaccharide (LPS) or, for non‐inflammatory induction, with staurosporine (STS) to trigger apoptosis. Organoids were dissociated into single cells with TrypLE Express and co‐cultured with macrophages at a 1:1 ratio. Macrophages were pre‐labeled with CMFDA (green) and organoid cells with CMTPX (red), and co‐culture was maintained for 1 h to allow efferocytosis. Apoptosis was assessed by Annexin V‐FITC/7‐AAD staining, and efferocytosis was quantified as the proportion of CMFDA/CMTPX double‐positive cells. Flow cytometry was performed on a BD LSR II (BD Biosciences, USA) and analyzed with FlowJo (Tree Star, USA). Gating strategies included exclusion of debris and doublets, live/dead discrimination, and quantification of apoptotic (Annexin V⁺/7‐AAD⁺) and efferocytic (CMFDA⁺/CMTPX⁺) populations.

### Immunofluorescence Staining

Paraffin‐embedded mouse colon sections were deparaffinized, rehydrated, subjected to antigen retrieval, blocked, and incubated with primary antibodies (1:100) overnight at 4 °C, followed by fluorescent secondary antibodies. For colonic organoids, apoptosis was induced with LPS and detected with an Annexin V‐FITC/7‐AAD kit. For cellular assays, macrophages were labeled with CMFDA (green) and organoids with CMTPX (red), dissociated with TrypLE, resuspended in medium, and co‐incubated for 1 h; Hoechst was used for nuclear staining. Imaging of both fixed and live samples was performed on a Zeiss LSM‐800 confocal microscope (Carl Zeiss AG, Oberkochen, Germany).

### Chromatin Immunoprecipitation (ChIP) Assays

ChIP assays were conducted to explore the interaction between NR1D1 and the CD47 promoter in colonic organoids. Following formaldehyde fixation, the cross‐linked protein‐DNA complexes were isolated using a commercial ChIP kit, according to the manufacturer's protocol. The complexes were incubated overnight at 4 °C with gentle agitation, using either an NR1D1‐specific antibody or rabbit IgG as a negative control. After incubation, magnetic beads were added and allowed to bind for 2 h with gentle agitation to facilitate the capture of antibody‐bound complexes. The bead‐bound DNA was then eluted and subjected to PCR amplification using primers flanking the NR1D1 binding region on the human CD47 promoter. The primers used were 5′‐CGCACTGTGTAGAGGATT‐3′ (forward) and 5′‐GTGGCTGCTCTAATGGAG‐3′ (reverse). PCR products were analyzed via agarose gel electrophoresis and visualized under UV light to confirm the presence of the target DNA fragments.

### Luciferase Reporter Assays

Luciferase reporter assays were conducted utilizing the pGL4.11‐Basic vector, which was modified to incorporate either the wild‐type (WT) or mutated (Mut) promoter region of CD47. Both NR1D1‐knowdown cells and control cells were transfected with these engineered reporter vectors, in conjunction with the pRL Renilla luciferase as a control reporter. Dual‐luciferase activities were subsequently measured using a luminometer (Promega, Madison, USA). The assay quantifies the relative activities of Firefly and Renilla luciferases within a single sample, offering an internal control to normalize transfection efficiency and sample handling variability. The luminescence from the Renilla luciferase serves as an internal control, thereby facilitating the normalization of Firefly luciferase expression.

### Statistical Analysis

All numerical data are expressed as mean ± standard deviation. For single comparisons, an unpaired two‐tailed Student's t‐test was used; for multiple comparisons, one‐way ANOVA followed by Bonferroni's post hoc adjustment was applied. For non‐parametric distributions, the Mann–Whitney U test was used. A *P* value <0.05 was considered statistically significant. All analyses were performed using GraphPad Prism version 9.5 (GraphPad Software, USA).

### Ethics Approval and Consent To Participate

This study adhered to ethical standards, with all procedures approved by relevant committees. Animal experiments were conducted under the approval of the Animal Ethics Committee of Huazhong University of Science and Technology (Approval No. 2024‐4118). For human participants, ethical approval was obtained from the Independent Ethics Committee of Wuhan Union Hospital (Approval No. 2024‐0261‐02). The study was carried out in accordance with the principles set forth in the Declaration of Helsinki.

## Conflict of Interest

The authors declare no conflict of interest.

## Author Contributions

C.Y.D. performed the primary experiments, led the data analysis, and took the lead in drafting the manuscript. L.J.M., L.J.R., Z.X.P., F.L., and Y.Q. contributed to the development, care, and management of the mouse models throughout the study. R.L. and L.R.Z. conceptualized the study, provided overall direction and critical insights during the research, and played a major role in revising and finalizing the manuscript.

## Supporting information



Supporting Information

## Data Availability

The data that support the findings of this study are available from the corresponding author upon reasonable request.
