# Supplementary material for: Andrias Davidianus Peptide Hydrogel Enables Sustained SR9011 Release to Promote Efferocytosis and Alleviate Colitis
Source: Small. 2025 Oct 30;21(50):e09049. doi: 10.1002/smll.202509049 (PMC12710159; doi:10.1002/smll.202509049)
Supplement: Supplementary file 1 — Supporting Information [file SMLL-21-e09049-s001.docx]

**Supplementary Figures**

Supplementary Figure.1

**
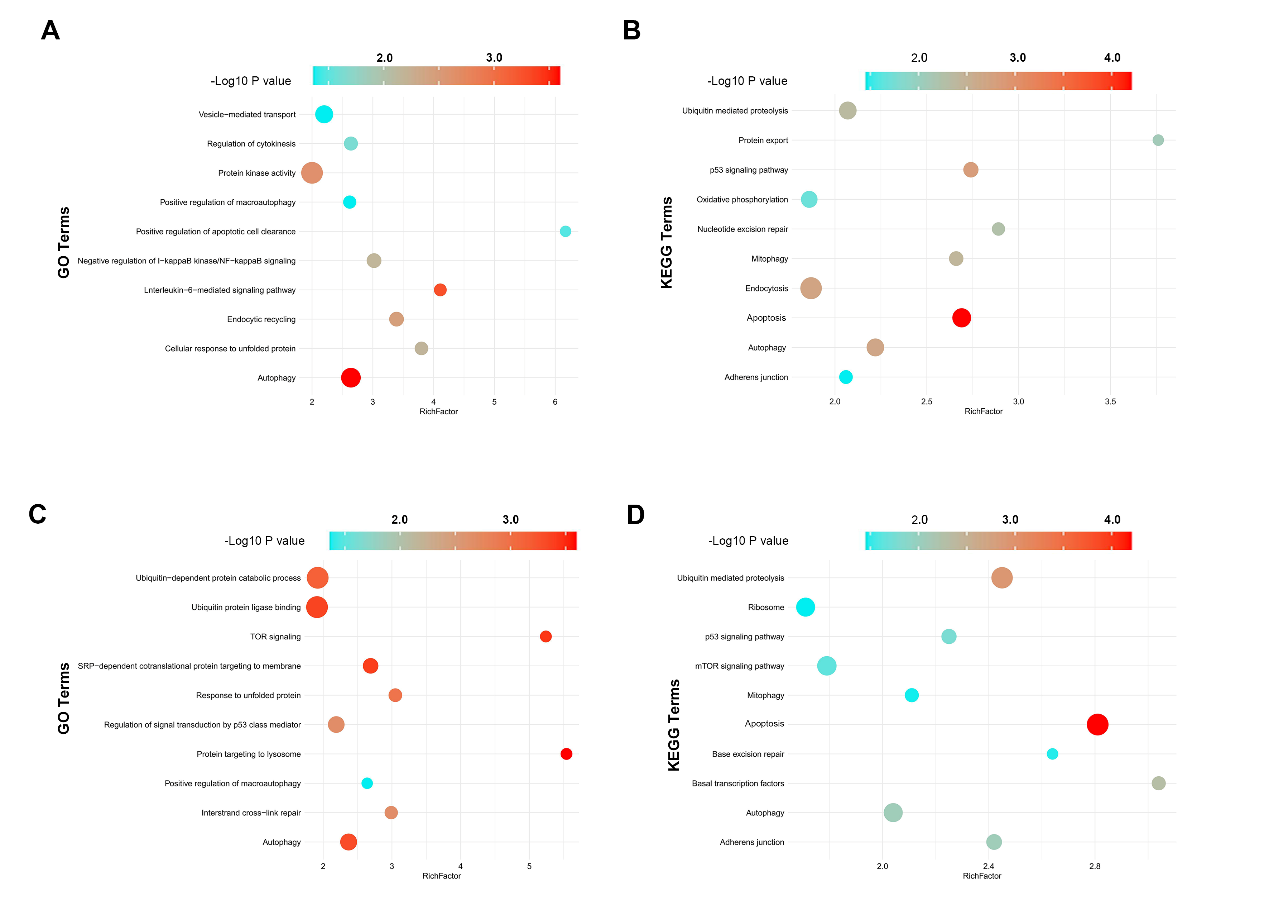
**

Supplementary Figure.2

**
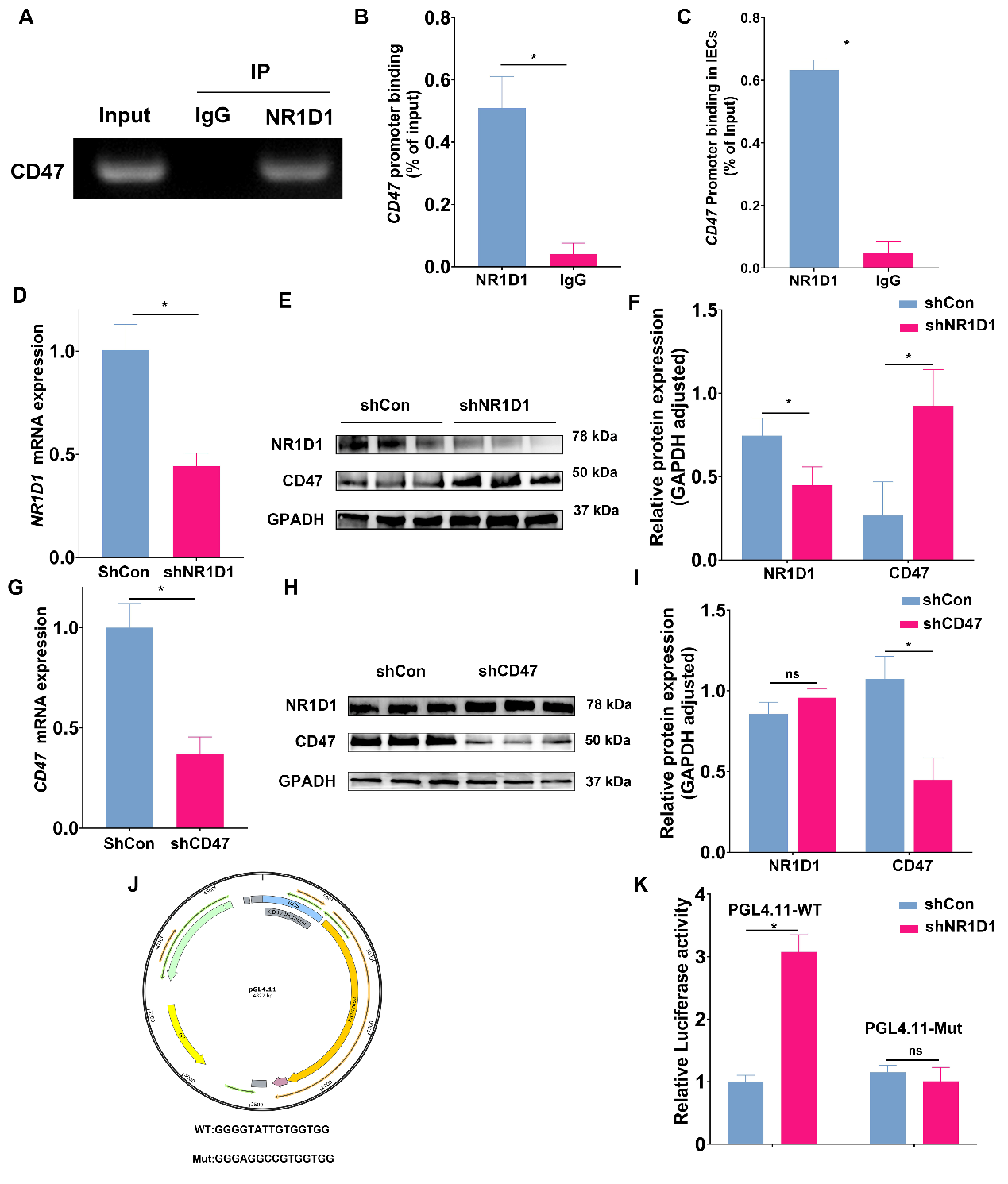
**

**
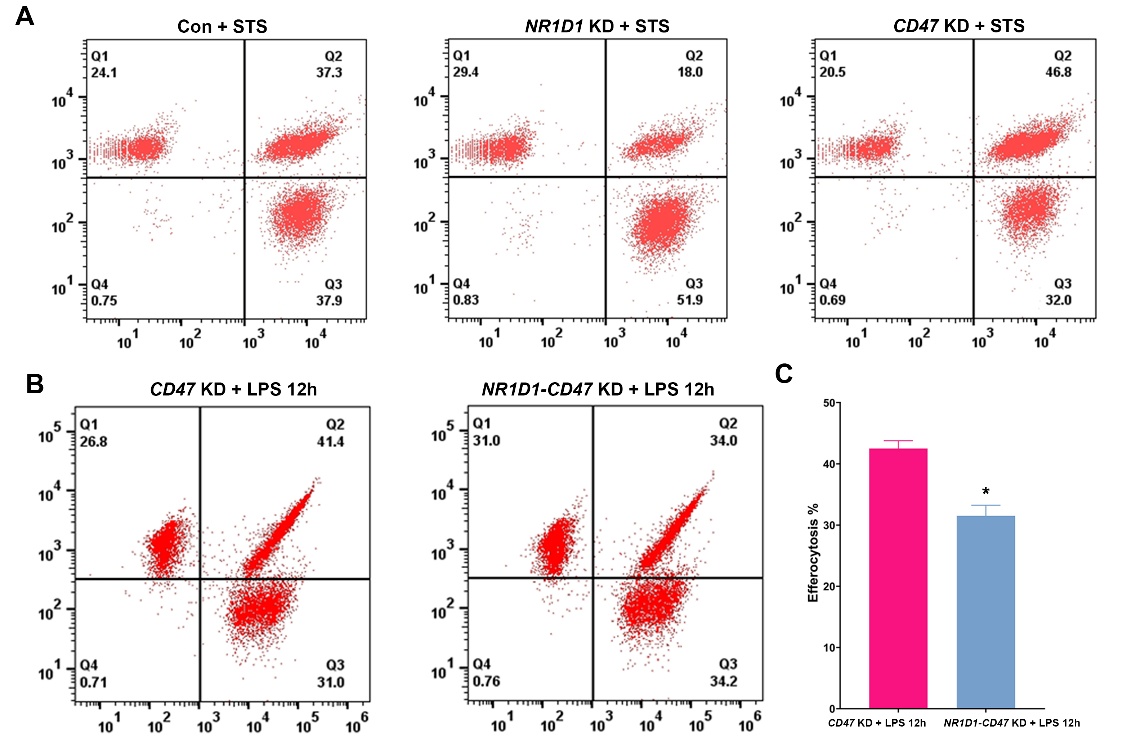
**Supplementary Figure.3

Supplementary Figure.4

**
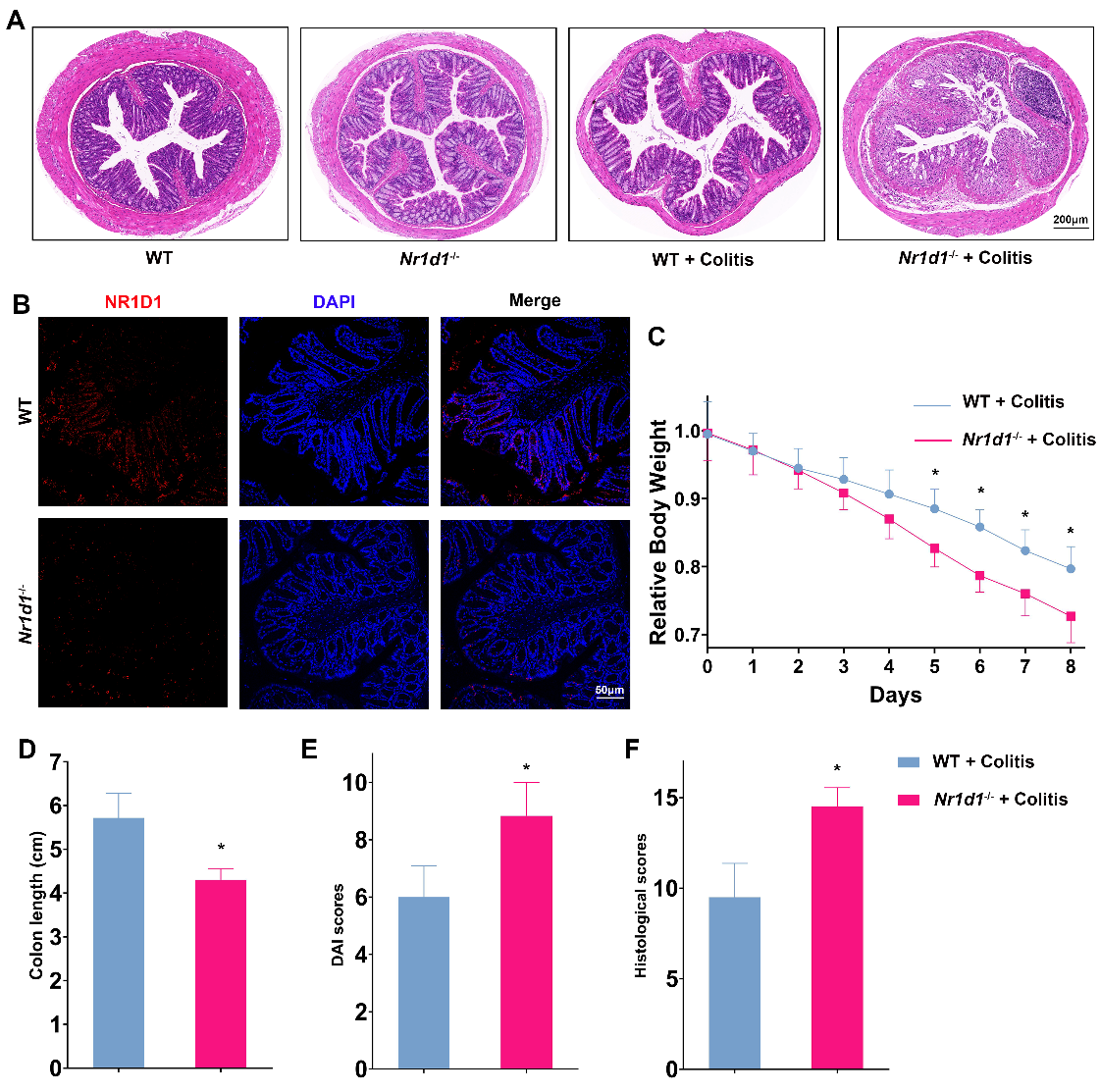
**

Supplementary Figure.5

**
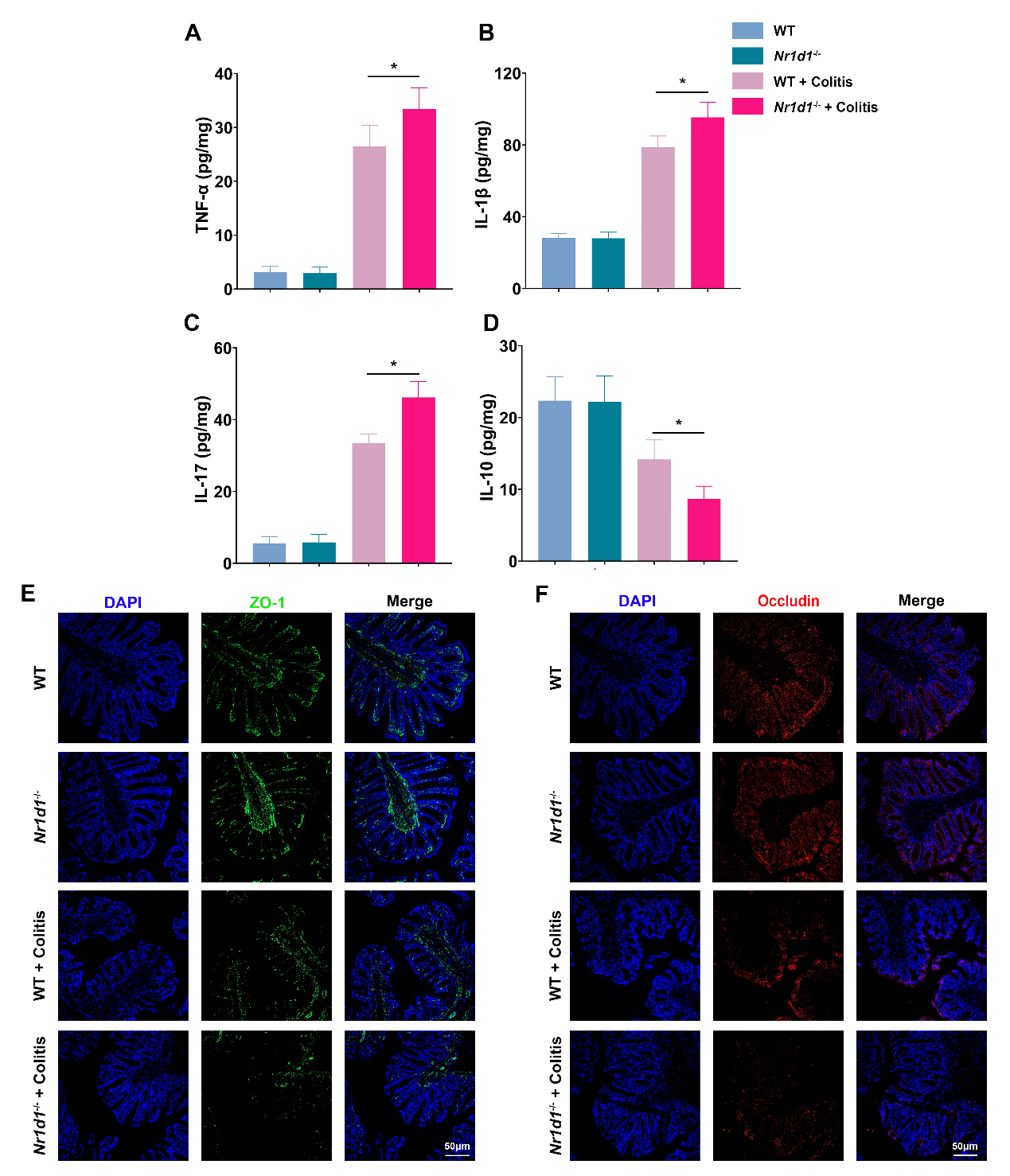
**

Supplementary Figure.6

**
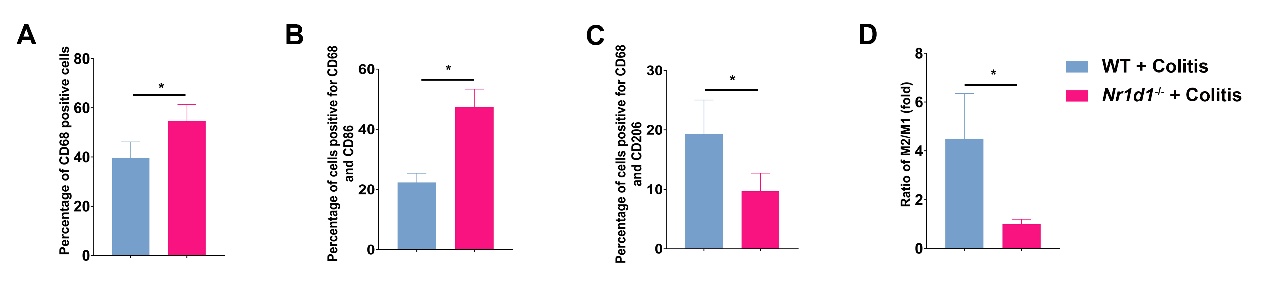
**

Supplementary Figure.7

**
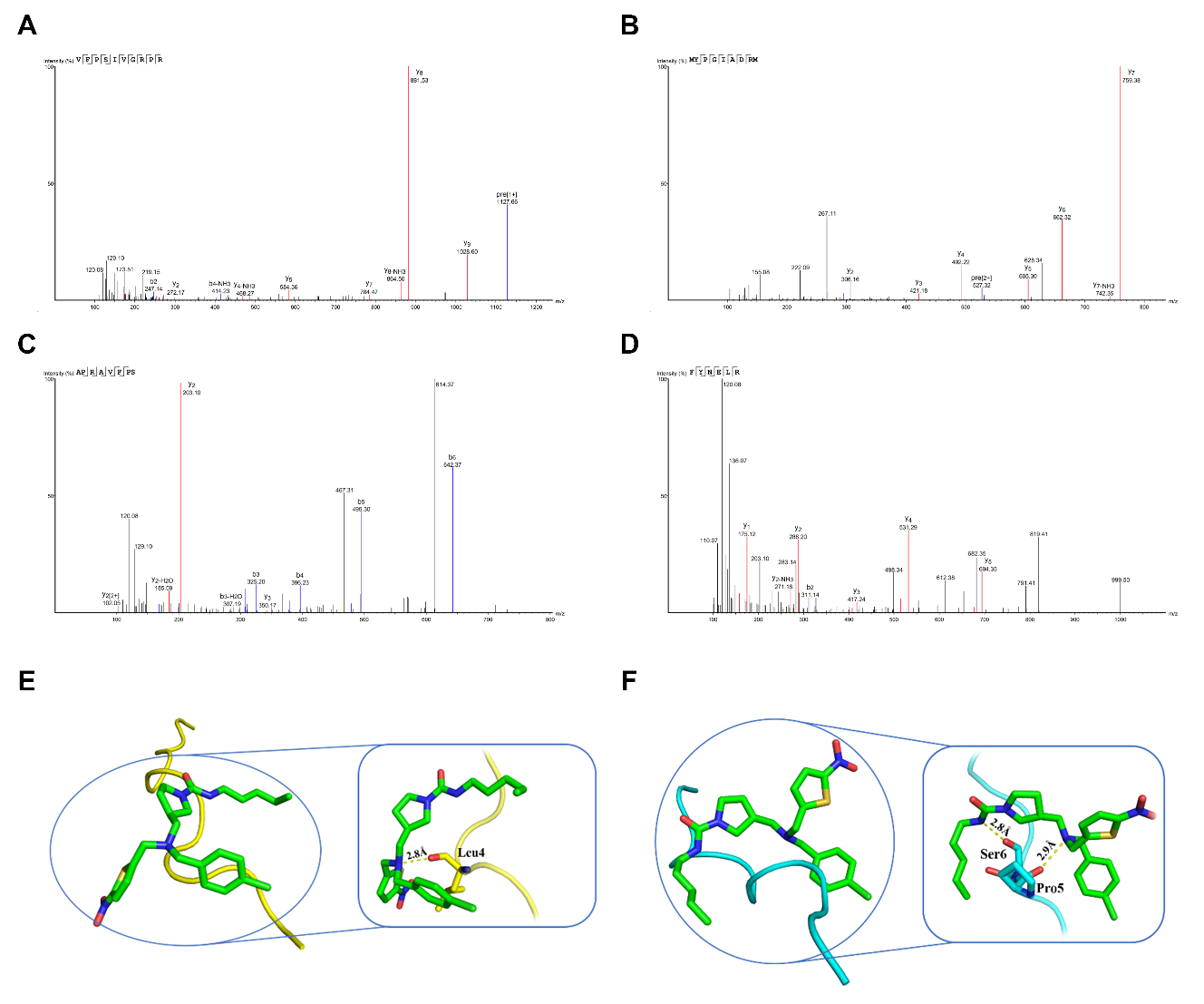
**

Supplementary Figure.8

**
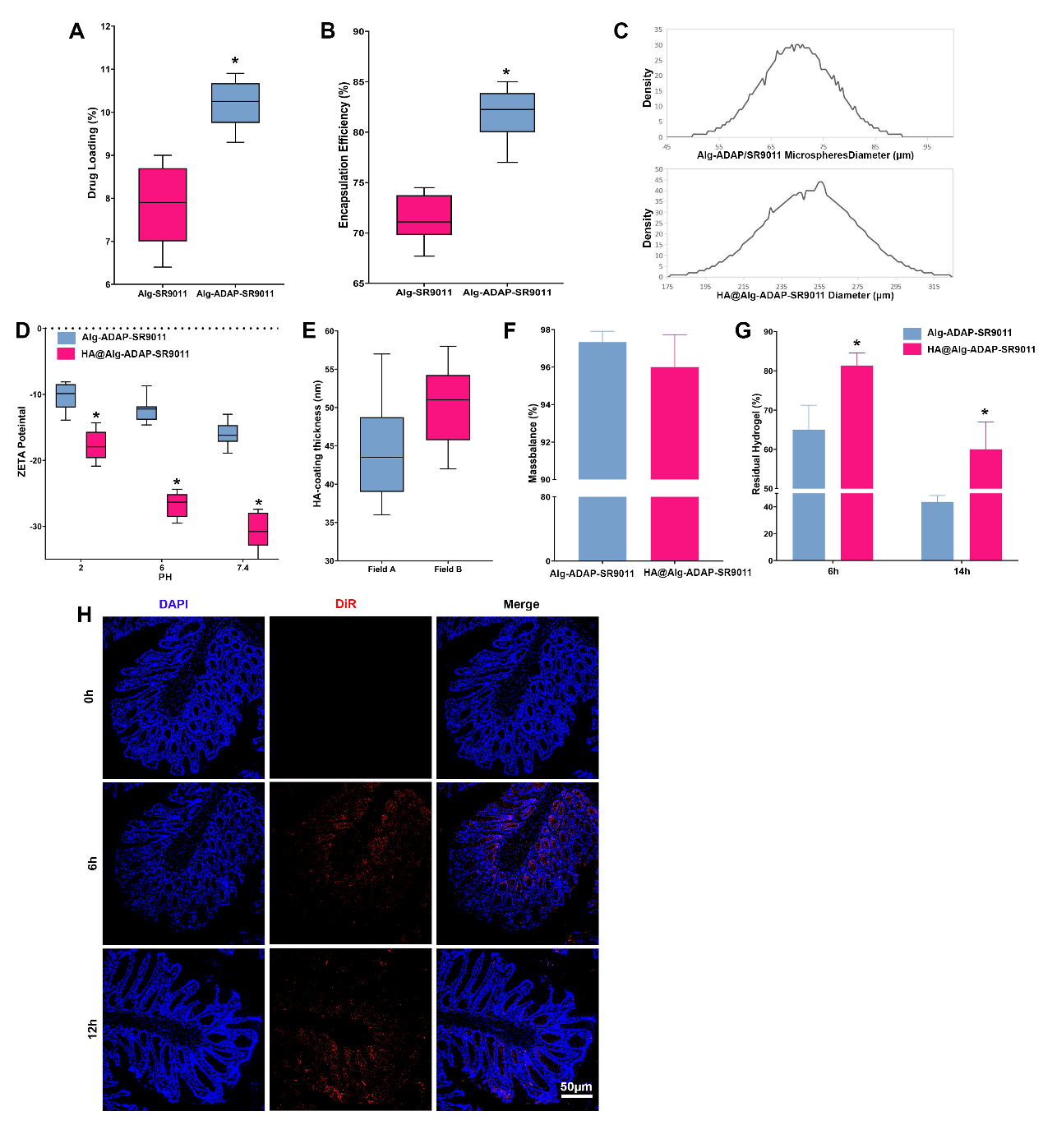
**

Supplementary Figure.9


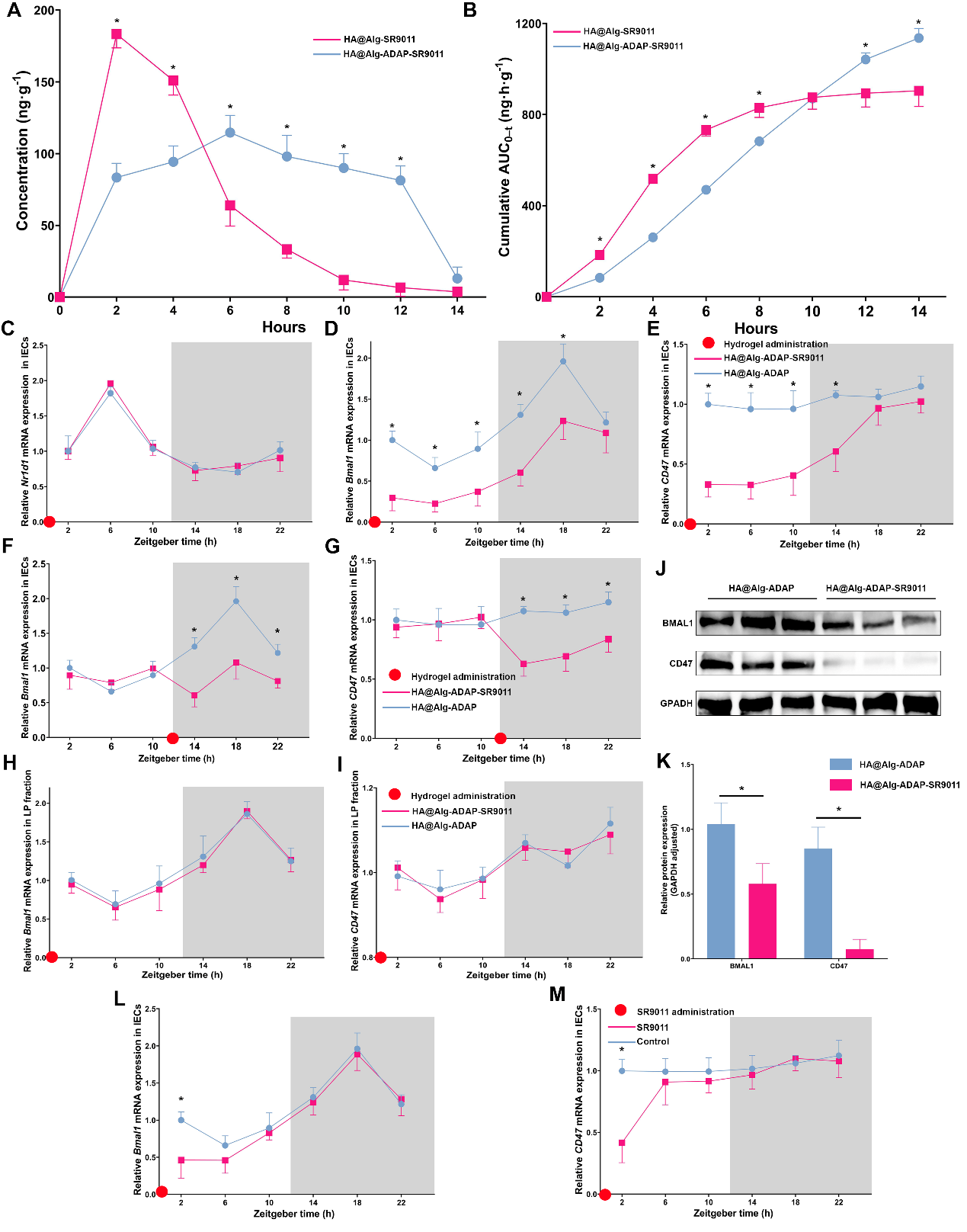


Supplementary Figure.10


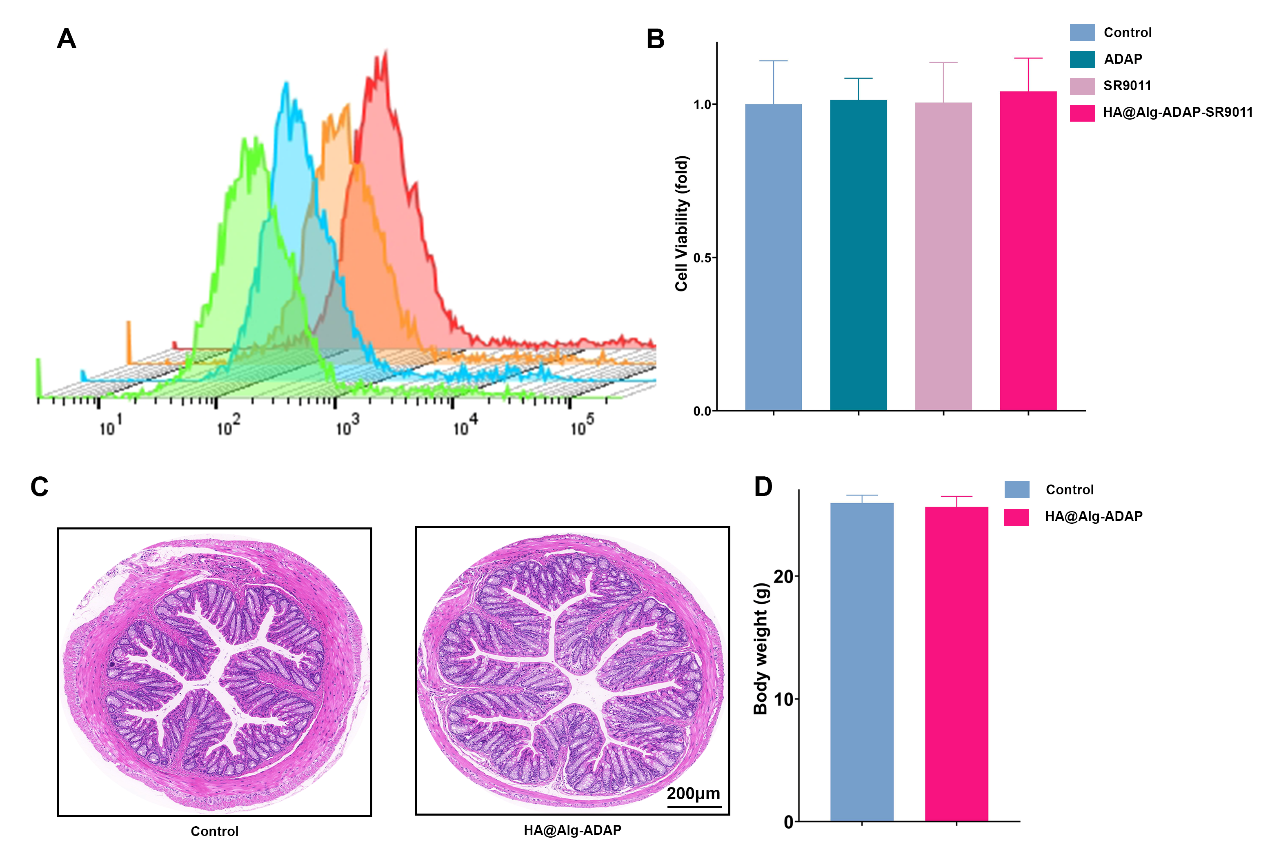


**Supplementary Figure 1 | GO and KEGG enrichment analyses of NR1D1 binding peaks in UC and control samples.**

(A, B) GO and KEGG enrichment of NR1D1 peaks unique to control mucosa, highlighting pathways related to efferocytosis, autophagy, cytokine regulation, and epithelial integrity. (C, D) GO and KEGG enrichment of NR1D1 peaks unique to UC mucosa, showing activation of stress-associated pathways, including mTOR, p53 signaling, and ubiquitin-mediated proteolysis. Circle size represents gene ratio; color denotes statistical significance (−log10 *P* value).

**Supplementary Figure 2 | NR1D1 occupancy at the CD47 promoter mediates transcriptional repression.**

(A, B) ChIP–qPCR in human colonic organoids showing NR1D1 enrichment at the CD47 promoter, with IgG as a negative control. (C) ChIP–qPCR confirming NR1D1 binding in mouse IECs. (D) qPCR validating efficient NR1D1 knockdown. (E, F) Western blot and densitometry demonstrating increased CD47 protein expression upon NR1D1 knockdown. (G) CD47 knockdown did not affect NR1D1 mRNA expression. (H, I) Western blot and quantification confirming unidirectional regulation, with CD47 knockdown having no effect on NR1D1 protein levels. (J) Schematic of luciferase reporter constructs carrying wild-type (WT) or NR1D1-binding site–mutant CD47 promoters. (K) Luciferase assay showing that NR1D1 knockdown enhanced CD47 promoter activity in the WT but not mutant construct, confirming direct transcriptional repression. Data are mean ± S.D.; unpaired t-test, **P* < 0.05; ns, not significant; n = 6 per group.

**Supplementary Figure 3 | NR1D1 and CD47 differentially regulate macrophage efferocytosis under inflammatory and non-inflammatory conditions.**

(A) Flow cytometry analysis of efferocytosis in macrophages co-cultured with apoptotic colonic organoids. Non-inflammatory apoptosis was induced with staurosporine; organoids were labeled with CMTPX (red) and macrophages with CMFDA (green). Conditions include control, NR1D1 knockdown, and CD47 knockdown. (B) Flow cytometry analysis of efferocytosis under LPS-induced apoptosis, comparing CD47 knockdown alone with combined NR1D1 and CD47 knockdown. (C) Quantification of efferocytosis from (B). Values are mean ± S.D.; unpaired t-test, **P* < 0.05; n = 6 per group.

**Supplementary Figure 4 | Epithelial-specific deletion of Nr1d1 exacerbates DSS-induced colitis.**

(A) Representative hematoxylin and eosin (H&E)–stained colonic sections from wild-type (WT), untreated *Nr1d1*^-/-^, and DSS-treated WT and *Nr1d1*^-/-^ mice. (B) Immunofluorescence staining showing NR1D1 expression in colonic epithelial cells of WT and *Nr1d1*^-/-^ mice. (C) Time-course analysis of relative body weight during DSS treatment. (D–F) Quantification of colon length (D), Disease Activity Index (DAI) scores (E), and histological inflammation scores (F), showing worse clinical and pathological outcomes in *Nr1d1*^-/-^ mice. Data are mean ± S.D.; unpaired t-test, **P* < 0.05; ns, not significant; n = 6 per group.

**Supplementary Figure 5 | NR1D1 deficiency promotes pro-inflammatory cytokine production and compromises intestinal barrier integrity during colitis.**

(A–D) ELISA analysis of colonic tissues showing increased levels of TNF-α, IL-1β, and IL-17 and decreased IL-10 in DSS-treated *Nr1d1*^-/-^ mice compared to wild-type (WT) controls. (E, F) Immunofluorescence staining of the tight junction proteins ZO-1 (green) and Occludin (red) in colonic epithelium. Data are mean ± S.D.; one-way ANOVA, **P* < 0.05; n = 6 per group.

**Supplementary Figure 6 | NR1D1 deficiency skews macrophage polarization and reduces M2/M1 ratio during colitis.**

(A) Quantification of CD68⁺ macrophages in the colonic mucosa of WT + Colitis and *Nr1d1*^-/-^ + Colitis mice. (B) Percentage of M1 macrophages (CD68⁺CD86⁺) showing increased pro-inflammatory polarization in *Nr1d1*^-/-^ mice. (C) Percentage of M2 macrophages (CD68⁺CD206⁺) demonstrating reduced anti-inflammatory, pro-efferocytic polarization in *Nr1d1*^-/-^ mice. (D) Quantitative analysis of the M2/M1 macrophage ratio, confirming a significant reduction in *Nr1d1*^-/-^ colitic mice. Data are mean ± S.D.; unpaired t-test, **P* < 0.05; ns, not significant; n = 6 per group.

**Supplementary Figure 7 | Mass spectrometry identification and molecular docking of ADAP peptides with SR9011.**

(A–D) Tandem mass spectrometry (MS/MS) spectra of representative Andrias davidianus active peptides (ADAP), showing annotated b- and y-ion series confirming the sequences VEPSIVGRPR (A), WYPGFADPM (B), APRAVFFPS (C), and TQNEFR (D). (E–F) Molecular docking simulations between ADAP-derived peptides and SR9011.

**Supplementary Figure** **8 | Physicochemical and functional characterization of HA@Alg-ADAP-SR9011 microspheres.**

(A) Drug loading (DL%) and (B) encapsulation efficiency (EE%) determined by mass-balance analysis. (C) Particle size distribution of alginate–ADAP–SR9011 droplets and HA-coated microspheres measured by laser diffraction. (D) ζ-potential at pH 2.0, 6.0, and 7.4. (E) HA-coating thickness quantified by atomic force microscopy. (F) In-vitro release and mass-balance analysis of SR9011 under simulated gastric, intestinal, and colonic conditions. (G) Mucoadhesion under physiologically relevant shear stress using excised porcine mucosa. (H) In-vivo residence and uptake visualized by fluorescence imaging of colonic sections at 0, 6, and 12 h after administration of DiR-labeled formulations. Data are mean ± S.D.; unpaired t-test, **P* < 0.05; ns, not significant; n = 6 per group.

**Supplementary Figure 9 |** **Pharmacokinetics and circadian regulation of SR9011 in vivo.**

(A, B) Colonic SR9011 concentrations and cumulative AUC after administration of HA@Alg-SR9011 or HA@Alg-ADAP-SR9011, measured by LC–MS/MS. (C–E) Nr1d1, Bmal1, and Cd47 mRNA expression in IECs under in-phase dosing at defined Zeitgeber times (ZT). (F, G) Bmal1 and Cd47 expression in IECs under out-of-phase dosing at ZT. (H, I) Bmal1 and Cd47 expression in lamina propria (LP) fractions under in-phase dosing. In the absence of spatial omics analyses, current evidence suggests that HA@Alg-ADAP-SR9011 predominantly affects IECs. (J, K) Western blot and densitometric quantification of BMAL1 and CD47 in IECs at ZT6. (L, M) Bmal1 and Cd47 expression in IECs following rectal administration of free SR9011. Data are mean ± S.D.; unpaired t-test, *P < 0.05; ns, not significant; n = 6 per group.

**Supplementary Figure** **10 | Biocompatibility and safety evaluation of ADAP-based formulations.**

(A) Flow cytometry analysis of reactive oxygen species (ROS) levels in colonic organoids exposed to Control, ADAP, SR9011, or HA@Alg-ADAP-SR9011 for 48 h. (B) Cell viability quantified by CCK-8 assay under the same conditions. (C) Representative H&E-stained colon sections from mice treated daily with HA@Alg-ADAP for 8 days versus untreated controls. (D) Body weight of mice after 8 days of treatment compared with controls. Data are mean ± S.D.; one-way ANOVA; n = 6 per group.

**Supplementary Table 1: Participant Demographics and Clinical Data from Wuhan Union Hospital**

| **Patient Characteristics** | **UC_Mayo ES 1** | **UC_ Mayo ES 2-3** | **Control** | **Remission** |
| --- | --- | --- | --- | --- |
| **Patients (n)** | 107 | 112 | 105 | 126 |
| Female | 53 | 51 | 54 | 67 |
| Male | 54 | 61 | 51 | 59 |
| Age (mean±SD)/year | 27.31 ± 5.23 | 25.94 ± 6.02 | 30.75 ± 4.75 | 31.46 ± 4.51 |
| Age range/year | 18-43 | 18-44 | 18-45 | 20-45 |
| **Mayo ES** |  |  |  |  |
| Mayo Score 0 | 0 | 0 | NA | 126 |
| Mayo Score 1 | 107 | 0 | NA | 0 |
| Mayo Score 2 | 0 | 59 | NA | 0 |
| Mayo Score 3 | 0 | 53 | NA | 0 |
| **Biopsy site** |  |  |  |  |
| Rectum | 107 | 112 | 105 | 126 |
